# A RAS(ON) Multi-Selective Inhibitor Combination Therapy Triggers Long-term Tumor Control through Senescence-Associated Tumor-Immune Equilibrium in Pancreatic Ductal Adenocarcinoma

**DOI:** 10.1158/2159-8290.CD-24-1425

**Published:** 2025-04-29

**Authors:** Caroline Broderick, Riccardo Mezzadra, Exequiel M. Sisso, Felix Mbuga, Rashi Raghulan, Almudena Chaves-Perez, Amanda Kulick, Lingyan Jiang, Jingjing Jiang, Yu-Jui Ho, Janelle Simon, Eric Rosiek, Eric Chan, Aveline Filliol, Ronan Chaligne, Elisa de Stanchina, Ximo Pechuan-Jorge, Andrea Schietinger, Mallika Singh, Scott W. Lowe

**Affiliations:** 1 Department of Cancer Biology and Genetics, Memorial Sloan Kettering Cancer Center, New York, New York.; 2 Weill Cornell Graduate School of Medical Sciences, New York, New York.; 3 Revolution Medicines, Redwood City, California.; 4 Anti-tumor Assessment Core Facility, Memorial Sloan Kettering Cancer Center, New York, New York.; 5 Molecular Cytology Core, Memorial Sloan Kettering Cancer Center, New York, New York.; 6 Single Cell Analytics Innovation Lab, Memorial Sloan Kettering Cancer Center, New York, New York.; 7 Department of Immunology, Memorial Sloan Kettering Cancer Center, New York, New York.; 8 Howard Hughes Medical Institute, Chevy Chase, Maryland.

## Abstract

Pharmacological inhibition of oncogenic RAS represents an attractive strategy to target pancreatic ductal adenocarcinoma (PDAC), an almost ubiquitously RAS-driven disease. However, initial responses to targeted monotherapy inhibition of active RAS can be followed by relapses, potentially driven by the persistence of drug-tolerant tumor cells. To target these “persister” cells, we investigated strategies to increase their immune visibility in mouse models of PDAC. We show that combining a RAS(ON) multi-selective inhibitor with the CDK4/6 inhibitor palbociclib drives persister cells into a senescent-like state, which coincides with improved tumor control and substantial remodeling of the tumor microenvironment. Combining RAS(ON) and CDK4/6 inhibition with a CD40 agonist results in durable regressions and CD4 T cell–dependent tumor-immune equilibrium. Our studies reveal a combinatorial approach that circumvents resistance to RAS(ON) inhibitor monotherapy in preclinical models and demonstrates a mechanism by which therapy-induced senescence can be reinforced by the immune system, resulting in durable tumor control.

**Significance:**

Our preclinical studies highlight an opportunity to exploit the senescence program and CD4 T cell–mediated mechanisms to achieve long-term tumor-immune equilibrium and control with RAS-targeted therapies. This work advances our understanding of therapy-induced senescence and suggests new avenues for combination therapies with the potential to benefit patients with PDAC.

*See related commentary by Lasse Opsahl and Pasca di Magliano, p. 1537*

## Teaser

Combining a multi-selective RAS(ON) inhibitor with CDK4/6 inhibition and CD40 agonism shifts drug-tolerant persister cells towards senescence and promotes durable tumor regression in pancreatic cancer.

## Introduction

Pancreatic ductal adenocarcinoma (PDAC) remains one of the most lethal cancers worldwide, with a dismal 5-year survival rate of around 10% (1, 2). PDAC is almost invariably initiated by activating mutations in the *KRAS* oncogene that, upon loss of tumor suppressors such as *TP53*, *CDKN2A*, and *SMAD4*, leads to the onset of highly metastatic tumors, which develop in a markedly immunosuppressive microenvironment. Standard-of-care interventions include surgery and conventional chemotherapies, but these rarely lead to durable responses. While molecularly targeted agents and immunotherapies have dramatically improved outcomes in certain cancer types, their potential has yet to be realized in patients with pancreatic cancer. Consequently, there is a pressing need for novel therapeutic strategies.

Oncogenic *KRAS* has long been considered a potential target in PDAC, first validated through studies relying on the genetic inactivation of this gene (3, 4). *KRAS*, along with its paralogs *NRAS* and *HRAS*, encodes GTPases that transmit growth factor signals from activated membrane receptors through intracellular signaling cascades, including the MAPK and PI3K pathways, which converge to promote cell growth and survival. Oncogenic *KRAS* mutations are dominated by missense mutations in three codons (12, 13, and 61) that drive KRAS into a constitutively active, GTP-bound state, thus leading to ligand-independent, aberrant growth signaling.

Recent advances in medicinal chemistry have enabled the development of small molecules targeting RAS (5). The earliest iterations of these inhibitors, which target KRAS^G12C^, have been approved for the monotherapy treatment of non–small cell lung cancer and, in combination with EGFR inhibition, for colorectal cancer. The majority of PDACs typically harbor *KRAS*^*G12D*^ or *KRAS*^*G12V*^ mutations, and various inhibitors directly targeting these mutant alleles, either selectively or via pan-RAS-selective agents, are currently being developed (6, 7). Additionally, RAS(ON) multi-selective tri-complex inhibitors capable of inhibiting both wild-type and mutant RAS in its active, GTP-bound state have shown robust single-agent activity in tumors driven by activating RAS mutations in preclinical studies (8–10), as well as in preliminary clinical studies in PDAC patients (11).

While RAS-targeted therapies generally have shown initial responses to monotherapy in clinical trials, the durability of such responses is limited by genetic and nongenetic resistance mechanisms (12–14). As occurs with other targeted therapies, initial responses to KRAS^G12D^-targeted and multi-selective RAS inhibitors are not durable, and cells that survive RAS inhibition, referred to here as “persister” cells, eventually evolve and acquire resistance mechanisms that give rise to relapse (10, 15–20). These findings underscore the need for combination regimens that target persister cells and may, in turn, maximize the clinical impact of these agents.

Like molecularly targeted therapies, immunotherapies represent another powerful treatment modality that has been difficult to harness in PDAC (21). Most current immunotherapy approaches involve immune checkpoint blockade (ICB), which has proven extraordinarily effective in some tumor types (e.g., lung cancer, melanoma) but has shown minimal activity in advanced PDAC, perhaps owing to PDAC’s low neoantigen load and/or its immunosuppressive environment (22–24). Nevertheless, a recent study demonstrated that an ICB agent combined with personalized, neoantigen-specific vaccines conferred durable responses in PDAC in the adjuvant setting (25). These studies indicate that engaging the immune system to target PDAC is possible and hint that such strategies may be most effective in contexts of low disease burden.

Another approach to enhance the immune recognition of PDAC involves the therapeutic induction of cell states that resemble senescence, a process that can be triggered by various cellular stressors (26). Senescent cells display a diminished proliferative capacity and adopt a “senescence-associated secretory phenotype” (SASP), characterized by increased expression of proinflammatory cytokines and other factors that can modulate the tissue environment in beneficial or detrimental ways (27–29). Though canonical senescence is dependent on tumor suppressors, such as p53 and p16INK4A, which are frequently lost in cancer (30), in established tumors, the genetic re-expression of tumor suppressors or treatments that induce DNA damage or target cell-cycle progression can push tumor cells into senescent-like states (26, 31, 32), but whether these states retain the irreversibility associated with canonical senescence remains an open question. Nonetheless, in some cases, senescent-like states can trigger an SASP-dependent influx of T cells into the tumor (31, 32). Such T-cell infiltration is associated with a positive response to immunotherapy in patients (33), and accordingly, combined inhibition of the MAPK effectors MEK or ERK and CDK4/6 in preclinical PDAC models was shown to trigger tumor cell senescence (34) and SASP-dependent T-cell infiltration and to sensitize tumors to ICB (32, 34, 35).

MEK inhibitors have been previously evaluated in the context of PDAC and exert little tumor control as monotherapies in mouse models thereof and, in KRAS mutant settings, are primarily cytostatic rather than cytotoxic (36, 37). In contrast, direct RAS inhibitors such as the KRAS^G12D^ allele–specific inhibitor MRTX-1133 or the RAS(ON) multi-selective inhibitor RMC-7977 induce rapid and potent tumor regressions and trigger substantial cell death (8–10, 17). We, therefore, examined whether the antitumor activity of RAS inhibition could be further potentiated by CDK4/6 inhibition and, if so, whether senescence biology plays any role in this response. Our results identify a combination regimen that is effective in prolonging therapeutic responses to RAS inhibition by immune-mediated reinforcement of tumor cells in a senescent-like state and indicate that clinical evaluation of this combination would be warranted.

## Results

### CDK4/6 Inhibition Enhances the Antitumor Activity of the RAS(ON) Multi-selective Inhibitors

**Figure 1.**
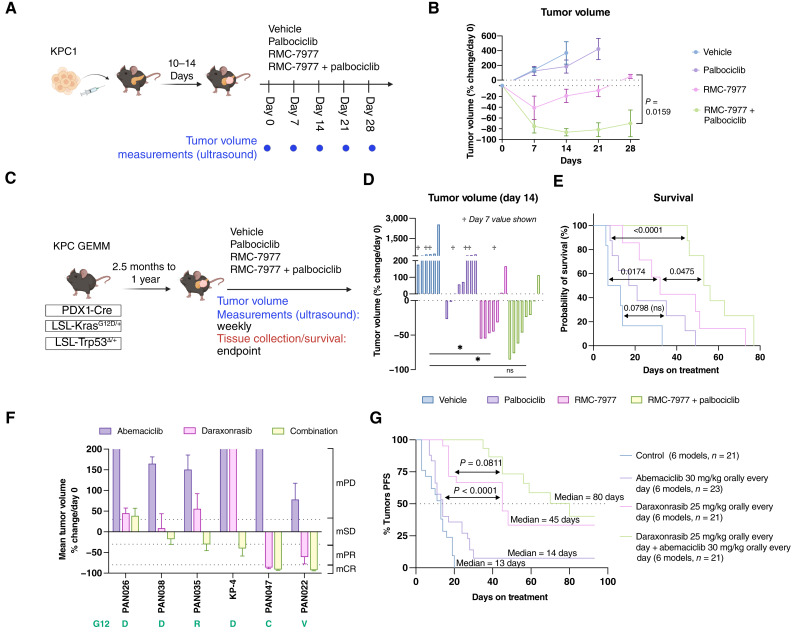
The CDK4/6 inhibitor palbociclib increases the antitumor activity of the RAS inhibitor in mouse models of PDAC. **A,** Scheme of experimental design (KPC1 orthotopic transplant into wild-type C57Bl/6 mice). **B,** Percentage change in tumor volume compared with day 0 as measured by weekly ultrasound. The *y*-axis indicates the percentage change in tumor volume, and the *x*-axis indicates the time point after treatment initiation. Each line represents the treatment group average. Statistical analysis: A two-tailed Mann–Whitney test was performed to compare the percentage change in tumor volume between the remaining RMC-7977-treated (*n* = 4) and RMC-7977 + palbociclib–treated (*n* = 5) mice at day 28. The *P* value is shown. **C,** Scheme of experimental design (KPC GEMM model). **D,** Percentage change in tumor volume compared with day 0 at 14 days after treatment initiation in KPC GEMMs following treatment with indicated agents. In mice that did not survive until day 14 ultrasound measurement, day 7 measurements are shown (indicated by a cross). Statistical testing: Ordinary one-way ANOVA, comparing the mean of each treatment group with the mean of every other treatment group and correcting for multiple comparisons with the Tukey test. Only statistically significant comparisons or relevant comparisons are shown. Sample size: vehicle (*n* = 6), palbociclib (*n* = 8), RMC-7977 (*n* = 7), RMC-7977 + palbociclib (*n* = 8). **E,** Probability of survival (%) in KPC GEMMs following treatment with indicated agents. Statistical testing: A log-rank (Mantel–Cox) test was performed to compare survival curves, and *P* values are shown. Sample size: vehicle (*n* = 6), palbociclib (*n* = 8), RMC-7977 (*n* = 7), RMC-7977 + palbociclib (*n* = 8). **F,** Percentage mean tumor volume change from day 0 at 21 days after treatment initiation in various CDX and PDX models. The mRECIST score was determined based on percentage mean tumor volume change, where mCR > 80% regression, mPR = 30%–80% regression, mSD = 30% regression – 30% growth, and mPD > 30% growth. Statistical testing: A two-way ANOVA with multiple comparisons, comparing the mean of each treatment group within each model shown and correcting for multiple comparisons with a Tukey test. Only daraxonrasib + abemaciclib (combo) versus abemaciclib or daraxonrasib in KP-4 was statistically significant (*P* < 0.0001 for both comparisons). The number of mice per treatment group per model is shown in **G**. **G,** Kaplan–Meier plot of progression-free survival in various CDX and PDX models, where progression is defined by tumor volume doubling from baseline. Statistical testing: A log-rank (Mantel–Cox) test was performed to compare survival curves, and *P* values are shown. Models are pooled, and the number of mice per treatment group is shown in the figure. (**A** and **C,** Created with BioRender.com.)

To investigate whether the antitumor activity of active RAS inhibition can be enhanced by CDK4/6 inhibition, we examined the RAS(ON) multi-selective inhibitor RMC-7977 and the CDK4/6 inhibitor palbociclib as single agents or in combination in orthotopic, autochthonous, and xenograft model systems. The orthotopic models utilize cell lines derived from tumors arising in two independent *Pdx1-Cre*, *LSL-Kras*^*G12D/+*^, and *LSL-Trp53*^*R172H/+*^ mice bearing primary pancreatic tumors [designated KPC1 and KPC2 (32)] that were subsequently engrafted into the pancreata of C57Bl/6 mice (Fig. 1A; Supplementary Fig. S1A). The autochthonous model involves strain intercrossing to produce a *Pdx1-Cre*, *LSL-Kras*^*G12D/+*^, and *LSL-Trp53*^*∆/+*^ genetically engineered mouse model (designated KPC GEMM), which faithfully recapitulates many of the histological and molecular features of human PDAC (38). In both model systems, mice were assigned to treatments using stratified randomization based on baseline tumor volume measurement (determined by ultrasound) and then treated with vehicle, palbociclib, RMC-7977, or RMC-7977 + palbociclib (Fig. 1A–E; Supplementary Fig. S1A and S1B). Changes in tumor size were monitored by ultrasound weekly for 28 days in the orthotopic model, at which point mice were euthanized. In the KPC GEMM model, mice were treated until humane endpoints as an indicator of overall survival (Fig. 1C–E).

Consistent with prior work (9, 10), RMC-7977 treatment in the orthotopic models led to rapid tumor regressions though tumors began to relapse by day 14 of treatment (Fig. 1B; Supplementary Fig. S1B). While palbociclib was ineffective as a single agent, the combination of RMC-7977 with palbociclib produced more durable regressions than those achieved with RMC-7977 alone (Fig. 1B; Supplementary Fig. S1B). With this combination, marked regressions were sustained through treatment day 28, a time when tumors treated with RMC-7977 had fully relapsed to baseline measurements (Fig. 1B). Similar results were observed in the KPC GEMM model, with the combination of palbociclib and RMC-7977 producing a trend toward enhanced tumor regressions compared with RMC-7977 alone at the 14-day time point (Fig. 1D) and resulting in a statistically significant improvement in overall survival (Fig. 1E). Therefore, palbociclib enhances the depth and durability of the antitumor activity of a RAS(ON) multi-selective inhibitor in both transplantable and autochthonous murine models of PDAC.

Consistent with this finding, daraxonrasib (RMC-6236), a similar RAS(ON) multi-selective inhibitor that is currently being evaluated clinically (9), showed combinatorial activity with the CDK4/6 inhibitor abemaciclib in a panel of cell line and patient-derived PDAC xenograft models. This manifested as a trend toward a more pronounced tumor growth inhibition or regression following 28 days of treatment with the combination compared with either daraxonrasib or abemaciclib alone in the PAN038, PAN035, and PAN022 models, and a statistically significant improvement in the KP-4 model (Fig. 1F). Importantly, the durability of treatment response – assessed for up to 90 days of treatment – was also improved, as indicated by a delayed time to tumor doubling with the combination compared with either monotherapy across models (Fig. 1G). Thus, the addition of CDK4/6 inhibition can improve the antitumor activity of RAS inhibition in human PDAC models.

### Palbociclib Drives Persister Cells That Survive RAS Inhibition into a Senescent-Like State

Though we previously reported that MAPK signaling attenuation via MEK inhibition can synergize with CDK4/6 inhibition, in line with a prior report (8), comparing the tumor control imparted by RMC-7977 with that observed with trametinib, either as a monotherapy or in combination with palbociclib, revealed strikingly more dramatic tumor regressions following RAS(ON) inhibition, suggestive of a distinct mechanism of action (Supplementary Fig. S1C).

**Figure 2.**
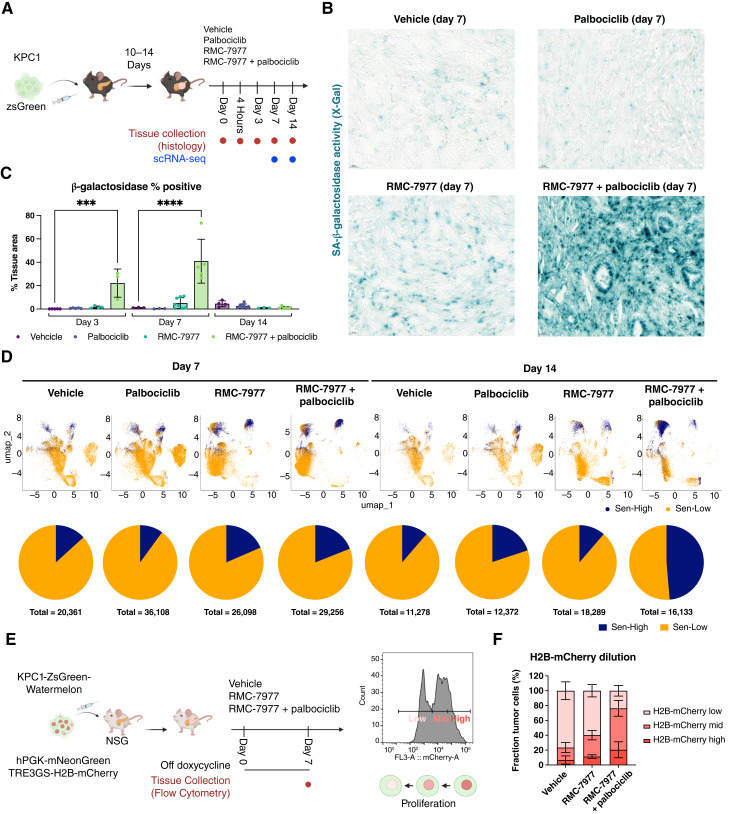
Combined RAS and CDK4/6 inhibition drives tumor cells into a senescence-like state. **A,** Scheme of experimental design for **B–D** and Supplementary Fig S3A–S3I (KPC1-ZsGreen orthotopic transplant into wild-type C57Bl/6 mice). **B,** Representative images of pancreatic tumor tissues following detection of β-galactosidase activity using the chromogenic substrate X-Gal. **C,** Quantification of IF staining of pancreatic tumor tissues for β-galactosidase activity in mice 4 hours, 3, 7, or 14 days after treatment initiation. Each dot represents an individual mouse (average of 3–5 ∼120,000 µm^2^ regions per mouse). Where data from two separate experiments were available, mice from experiments were merged (*n* = 3–8 mice per treatment group). Statistical testing: Ordinary one-way ANOVA, comparing preselected pairs of columns (within treatment time point vs. vehicle only) and correcting for multiple comparisons with a Bonferroni test. Only statistically significant comparisons are shown. **D,** Uniform Manifold Approximation and Projection for Dimension Reduction (UMAP) showing tumor cells from each treatment group classified as “Sen-High” in blue and those classified as “Sen-Low” in yellow. “Sen-High” cells are defined as those that are positive for three senescence signatures in Supplementary Fig. S3F–S3H). Pie charts below show the fraction of total cells classified as Sen-High versus Sen-Low in each treatment group. The number of sequenced tumor cells is displayed for each treatment group. **E,** Scheme of experimental design (KPC1-ZsGreen-Watermelon orthotopic transplant into NSG mice) to trace the proliferative history of tumor cells following the indicated treatments. The strategy for gating DAPI^−^, ZsGreen^+^ tumor cells based on H2B-mCherry levels is shown. **F,** Fraction of tumor cells with highly retained (stably arrested since treatment initiation), mid, or low H2B-mCherry following the indicated treatments. Statistical testing: Two-way ANOVA, comparing the mean of each treatment group within each H2B-mCherry with every other treatment group. Multiple comparisons testing was corrected for with a Tukey test, and statistically significant results are reported here. H2B-mCherry high: vehicle versus RMC-7977 + palbociclib (*P* = 0.0458). H2B-mCherry mid: vehicle versus RMC-7977 + palbociclib (*P* < 0.0001); RMC-7977 versus RMC-7977 + palbociclib (*P* < 0.0001). H2B-mCherry low: vehicle versus RMC-7977 (*P* = 0.0128); vehicle versus RMC-7977 + palbociclib (*P* < 0.0001); RMC-7977 versus RMC-7977 + Palbociclib *P* < 0.0001). (**A** and **E,** Created with BioRender.com.)

Tumor regressions triggered by RMC-7977 in murine PDAC have previously been linked to apoptosis (8, 10). To determine whether enhanced apoptosis or other mechanisms could account for the prolonged tumor responses with combined RMC-7977 and palbociclib, we assessed changes in tumor cell states upon treatment with these compounds as single agents and in combination. Since the RMC-7977 + palbociclib treatment produced similar effects in the autochthonous GEMM and the orthotopic transplantable models, we conducted mechanistic studies in the orthotopic model to enable rapid and synchronous tumor formation. C57Bl/6 mice were surgically implanted with KPC1 cells engineered to express the fluorescent protein ZsGreen (KPC1-ZsGreen) and allowed to engraft (Fig. 2A). Tumor-bearing mice were assigned to treatment groups by tumor volume and then treated with vehicle, palbociclib, RMC-7977, or RMC-7977 + palbociclib. Tumor tissue was collected at several time points after treatment initiation: 4 hours [the time of maximal p-ERK inhibition following the first dose of RMC-7977 (10)], 3 days (an early regression time point), 7 days (the maximum RMC-7977–induced regression), and 14 days (when profound combinatorial activity was observed; Fig. 1B, Supplementary Fig. S1B). At days 7 and 14 after treatment initiation, tumors were also digested and sorted for CD45^−^, ZsGreen^+^ tumor cells and processed for single-cell RNA sequencing (scRNA-seq; Fig. 2A).

As expected, immunofluorescence (IF) microscopy revealed elevated cleaved caspase 3 signal in tumors at 4 hours and 3 days following RMC-7977 treatment initiation, indicating that a substantial fraction of cells undergo cell death (Supplementary Fig. S2A and S2B). Notably, tumors from mice treated with RMC-7977 alone or RMC-7977 + palbociclib showed similar levels of cleaved caspase 3 staining, implying that palbociclib does not potentiate the antitumor activity of RMC-7977 by increasing the fraction of apoptotic tumor cells (Supplementary Fig. S2A and S2B). As reported, RMC-7977 treatment reduced phosphorylation of the RAS-RAF effector proteins ERK1/2 (p-ERK) at 4 hours following the first dose (reported t_max_), indicating inhibition of the RAS/MAPK pathway (Supplementary Fig. S2C and S2D; ref. 10). While adding palbociclib did not potentiate apoptosis or p-ERK inhibition, the tumor cells that survived treatment showed greater senescence-associated (SA)-β-galactosidase activity at 3 and 7 days after treatment initiation, a marker that preferentially accumulates in cells with features of the senescence program(Fig. 2B and C; ref. 39).

To further investigate the combinatorial activity of RMC-7977 and palbociclib and the potential involvement of senescence-related programs, we assessed transcriptional changes by analyzing scRNA-seq data collected from sorted tumor cells treated with vehicle, either of the single agents or the combination. Data were processed using the Seurat pipeline (40), followed by clustering to identify six cell clusters with distinct transcriptional states (Supplementary Fig. S3A and S3B). Tumor cells from vehicle- and palbociclib-treated tumors showed a high degree of overlap in cluster distribution at day 7; however, at day 14, an increase in clusters 2 and 3 and a decrease in cluster 5 were observed (Supplementary Fig. S3C). RMC-7977 treatment, alone or in combination with palbociclib, profoundly altered the frequencies of cells residing in each cluster. Notably, a striking reduction of cells residing in the *Myc*-high, proliferative cluster 2 was observed at both days 7 and 14 after treatment initiation (Supplementary Fig. S3B and S3C), in line with a prior study in which genetic perturbation of *MYC* sensitizes tumor cells to palbociclib and is potentially suggestive of a mechanism by which RAS inhibition–induced depletion of *Myc*-expressing cells mimics this phenomenon (34). A transient depletion of cluster 4, enriched for several proliferation-related genes, including *Mki67*, *Top2a*, and *Ccna2*, was also observed at 7 days after treatment initiation (Supplementary Fig. S3B and S3C). Of note, both of these clusters (4 and 2), which were either durably or transiently diminished in response to RMC-7977, displayed the highest MAPK pathway activity scores from PROGENy (Supplementary Fig. S3D), likely rendering them most sensitive to MAPK disruption via RAS inhibition.

Intriguingly, a marked expansion of cells residing in cluster 1, high for genes encoding several collagens, as well as *Lgals7*, *Acta2*, *Crabp2*, and *Grem1*, was observed at day 14 after treatment initiation exclusively in the setting of the combination treatment (Supplementary Fig. S3B and S3C). This cluster exhibited the highest TGF-β, TNFα, NF-κB, Trail, and p53 PROGENy pathway activity scores (Supplementary Fig. S3D) and was further enriched for multiple Hallmark Gene Signatures associated with senescence, including epithelial–mesenchymal transition, a program associated with RAS inhibitor resistance (41) that is also observed in senescent states (42), angiogenesis (32), and immune cell modulation, including IL6/JAK/STAT3 signaling, TNFα signaling via NF-κB, and IFN-γ and IFN-α response signatures (Supplementary Fig. S3E). Notably, senescent cells are known to become hypersensitive to IFN-γ signaling, which enhances their capacity for antigen presentation (31).

We next explored whether we could detect cells enriched for transcriptional signatures linked to senescence, and if so, whether RMC-7977 + palbociclib treatment increased the fraction of these cells. To identify cells in senescent-like states, transcriptional levels of published senescence-related signatures were assessed across Seurat clusters (Supplementary Fig. S3E–S3G). Expression of these signatures was highest in clusters 1 and 3 (Supplementary Fig. S3E–S3G), clusters in which most cells were classified as being in G_1_ of the cell cycle (Supplementary Fig. S3H). Thresholding of “high” versus “low” expression for “SenMayo,” “Senescopedia_Secreted,” and “SASP” was determined by performing a Gaussian mixture model, and “Sen-High” cells were defined as those that were high for expression of each of these three signatures (Fig. 2D; Supplementary Fig. S3E–S3H), which mainly resided within clusters that scored as G_1_ (Supplementary Fig. S3I). Tumors treated with RMC-7977 monotherapy showed a slight, transient increase in the fraction of cells assuming a senescent-like state at day 7 that was diminished by day 14, indicative of escape from incomplete senescence induction or outgrowth of cells that failed to engage this program (Fig. 2D). In contrast, tumor cells treated with RMC-7977 + palbociclib displayed an enrichment of senescent-like cells, around 50% of which resided in these clusters at day 14 (Fig. 2D). Intriguingly, SA-β-galactosidase was diminished at this time point, suggesting that some hallmarks of the senescence-like phenotype may peak earlier than day 14 but that tumor cells retain expression of senescence-related genes (Fig. 2B and C). Altogether, these analyses reveal that the profound antitumor activity imparted by combined RMC-7977 + palbociclib treatment coincides with the accumulation of tumor cells in a senescent-like state.

A hallmark of senescent-like states is their more durable proliferative arrest. To functionally investigate whether combined RMC-7977 and palbociclib treatment triggered a more stable cell-cycle arrest *in vivo*, we incorporated the Watermelon vector system, which consists of a doxycycline-inducible H2B-mCherry transgene (13), where the mCherry reporter is linked to H2B, a core histone protein, to trace the proliferative history of tumor cells following treatment. Tumor cells are labeled with H2B-mCherry induced by doxycycline addition, and following doxycycline withdrawal, mCherry fluorescence is diluted upon proliferation but retained in nondividing cells. We, therefore, tested whether combined treatment with RMC-7977 + palbociclib would lead to greater mCherry retention compared with treatment with vehicle or monotherapy RMC-7977.

To mitigate potential immunogenicity that the Tet-ON 3G (rtTA) element might introduce, KPC1-ZsGreen cells expressing the Watermelon vector were orthotopically transplanted into NOD-*scid* IL2Rg^null^ (NSG) mice (Fig. 2E) or CAGs-rtTA3 mice (Supplementary Fig. S3J) fed a doxycycline diet to induce the H2B-mCherry transgene (Fig. 2E). Upon tumor formation, doxycycline was withdrawn, and mice were treated with vehicle, RMC-7977, or RMC-7977 + palbociclib. Seven days later, tumor cell levels of H2B-mCherry were analyzed by flow cytometry, and cells were classified as having high (arrested), mid (late arrest or early bypass), or low (bypass) H2B-mCherry levels. Consistent with a more durable proliferative arrest, tumor cells derived from mice treated with RMC-7977 + palbociclib retained substantially more H2B-mCherry than those treated with vehicle or RMC-7977 alone (Fig. 2F). Importantly, this H2B-mCherry retention persisted when doxycycline was withdrawn from day 7 until day 14 after treatment initiation (Supplementary Fig. S3J and S3K) when, despite the observed decrease in SA-β-galactosidase at this time point (Fig. 2B and C), transcriptional states associated with senescence were enriched (Fig. 2D). These findings support a model whereby palbociclib drives tumor cells that survive RMC-7977 treatment into a senescence-like state.

### Palbociclib and RMC-7977 Synergize to Produce an Inflamed Tumor Microenvironment

**Figure 3.**
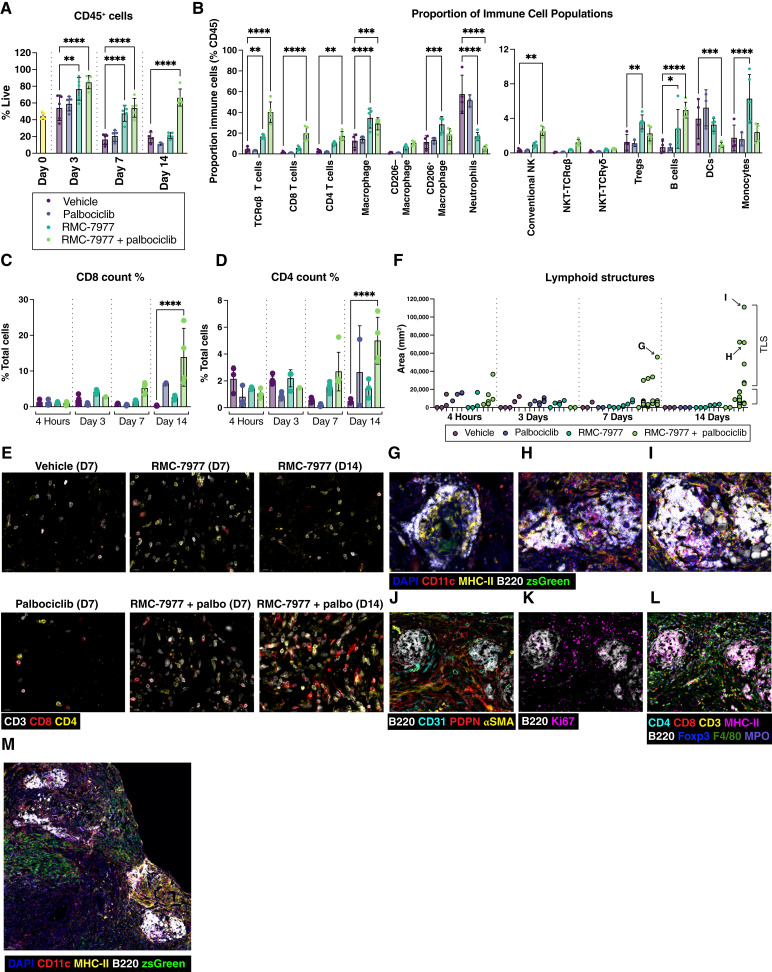
Combined RASi + CDK4/6i triggers favorable immune remodeling and poises tumors to respond to immunotherapy. For the scheme of experimental design, see Supplementary Fig. S5A (KPC1-zsGreen orthotopic transplant into wild-type C57Bl/6 mice). **A,** Fraction of CD45 cells out of total live cells by flow cytometry. Each dot represents an individual mouse. Statistical testing: Ordinary one-way ANOVA with multiple comparisons, comparing the means of each treatment group against the vehicle of the relevant time point and correcting for multiple comparisons using a Sidak test. Only statistically significant comparisons are shown. **B,** Fraction of indicated immune cell populations (%) out of total CD45^+^ immune cells at day 14 after treatment initiation. Each dot represents an individual mouse. Statistical testing: Two-way ANOVA with multiple comparisons, comparing the means of each treatment group against the vehicle and correcting for multiple comparisons using a Dunnett test. Only statistically significant comparisons are shown. **C,** Quantification of representative regions of IF staining for CD4 T cells (representative images shown in **E**) in tumors 4 hours, 3, 7, or 14 days after treatment initiation. Each dot represents an individual mouse (average of 3–5 ∼40,000 µm^2^ regions). Statistical testing: Ordinary one-way ANOVA with multiple comparisons, comparing the means of each treatment group against the vehicle at the relevant time point and correcting for multiple comparisons using a Bonferroni test. Only statistically significant comparisons are shown. **D,** Quantification of representative regions of IF staining for CD8 T cells (representative images shown in **E**) in tumors 4 hours, 3, 7, or 14 days after treatment initiation. Each dot represents an individual mouse (average of 3–5 ∼40,000 µm^2^ regions). Statistical testing: Ordinary one-way ANOVA with multiple comparisons, comparing the means of each treatment group against the vehicle of the relevant time point and correcting for multiple comparisons using a Bonferroni test. Only statistically significant comparisons are shown. **E,** Representative images of IF staining for T-cell markers CD3 (white), CD8 (red), and CD4 (yellow) following indicated treatments at indicated time points (D7 = 7 days after treatment initiation, D14 = 14 days after treatment initiation). **F,** Quantification of the number and area (µm^2^) of lymphoid aggregates (clusters of B cells, DCs, and other MHC-II positive cells) found in whole-slide scans of tumors. Each tick on the *x*-axis represents a single mouse. Each dot represents an individual lymphoid aggregate, and the size of each aggregate is reflected on the *y*-axis. The color of each dot indicates the treatment group, as shown in the figure legend. Arrows and corresponding letters point to lymphoid aggregates for which examples are shown in **G–I**. **G,** Example of a lymphoid aggregate from an RMC-7977 + palbociclib–treated mouse at *t* = 7 days after treatment initiation. **H** and **I,** Example of a lymphoid aggregate from an RMC-7977 + palbociclib–treated mouse at *t* = 14 days after treatment initiation. **J,** Highly multiplexed IF images of TLSs in tumors from mice treated with RMC-7977 + palbociclib for 7 days. Images were acquired by the Cell Dive and stained for endothelial cell (CD31), fibroblast (Podoplanin: PDPN), and activated fibroblast (α-smooth muscle actin: αSMA) markers. **K,** Highly multiplexed IF images of TLSs in tumors from mice treated with RMC-7977 + palbociclib for 7 days. Images were acquired by the Cell Dive and stained for B-cell (B220) and proliferation (Ki67) markers. **L,** Highly multiplexed IF images of TLSs in tumors from mice treated with RMC-7977 + palbociclib for 7 days. Images were acquired by the Cell Dive and stained for the indicated markers. **M,** Low magnification image of lymphoid aggregates shown in **H** and **I** following 14 days of RMC-7977 + palbociclib treatment.

KRAS inhibition, tumor cell death, and therapeutic induction of senescence (via the SASP) have all been linked to immune remodeling (17, 43–45). We, therefore, profiled the immune composition of orthotopic KPC1-ZsGreen tumors at 0, 3, 7, or 14 days after treatment initiation (Supplementary Fig. S4A), the latter time point being when both senescence-related gene expression and proliferative arrest were observed (Fig. 2D; Supplementary Fig. S3J and S3K). Immunophenotyping by flow cytometry revealed that the CD45^+^ fraction of immune cells was elevated upon RMC-7977 monotherapy and RMC-7977 + palbociclib treatment compared with vehicle, with many immune cell populations showing significant changes at day 14 after treatment initiation (Fig. 3A; Supplementary Fig. S4B–S4N). At this time point, residual tumors following RMC-7977 + palbociclib treatment demonstrated elevated fractions of CD4 T cells, CD8 T cells, conventional NK cells, TCRαβ-positive Natural Killer T (NKT) cells, macrophages, B cells, and γδ T cells, which trended toward being more pronounced than those observed in tumors treated with either monotherapy (Fig. 3B; Supplementary Fig. S4C–S4E, S4G–S4I, S4L). RMC-7977 treatment, either alone or in combination, also resulted in a depletion of dendritic cells (DC) and neutrophils (Fig. 3B; Supplementary Fig. S4K and S4N). Therefore, RMC-7977 + palbociclib promotes substantial immune remodeling at day 14 after treatment initiation.

Multiplexed IF staining of tumor tissues after treatment confirmed enhanced CD4 and CD8 T-cell infiltration upon treatment with the RMC-7977 + palbociclib combination at 14 days after treatment initiation (Fig. 3C–E). Strikingly and exclusively in the combination context, we observed the emergence of immune cell clusters reminiscent of tertiary lymphoid structures (TLS; Fig. 3F–L), which are observed in settings of chronic inflammation (46). As has been described for bona fide TLSs in other contexts (47), these immune cell clusters were adjacent to high endothelial venules (Fig. 3J) and contained CD11c^+^MHCII^+^ cells and a central zone of B220^+^ B cells (Fig. 3G–I), some of which were positive for the proliferation marker Ki67 (Fig. 3K). These structures were surrounded by CD4 T cells and F4/80^+^ macrophages (Fig. 3L), both of which can serve as lymphoid tissue inducer cells (46). Importantly, these immune cell clusters were observed within tumor borders, suggesting that they are not merely expanded pancreatic lymph nodes that are adjacent to the tumor (Fig. 3M).

Given the observed increase in T cells, B cells, and NK cells upon RMC-7977 + palbociclib treatment (Fig. 3B), all of which have been shown to be capable of exerting antitumor immunity, we assessed their role in the combinatorial antitumor activity of RMC-7977 + palbociclib. Despite substantial evidence of the expansion of these immune cell subsets following RMC-7977 + palbociclib treatment, responses were fairly comparable between immune-competent C57Bl/6 mice and immune-deficient NSG mice, which lack T cells, B cells, and NK cells and are deficient and defective in myeloid populations, at least for the 28 days of treatment assessed in this model (Supplementary Fig. S4O and S4P). In line with a lack of T-cell cytotoxic activity, CD8 T cells had diminished levels of the cytotoxic effector molecule granzyme B in response to RMC-7977 and RMC-7977 + palbociclib treatment (Supplementary Fig. S4Q and S4R). Thus, the initial antitumor control imparted by RMC-7977 + palbociclib treatment appears largely cell-intrinsic.

Despite this apparent lack of T cell–mediated tumor surveillance, a substantial fraction of the CD8 and CD4 T cells present in tumors treated with either RMC-7977 or RMC-7977 + palbociclib were proliferative (Ki67-positive) and expressed CD44, suggesting that these tumor cells are activated and antigen-experienced, indicative of tumor reactivity (Supplementary Fig. S4Q and S4R). Therefore, the observed lack of T-cell activity is unlikely to be a consequence of insufficient tumor recognition. A high fraction of T cells from tumors across all treatment groups stained positive for the exhaustion marker PD-1, suggesting that T-cell dysfunction through PD-1 signaling may play a role in inhibiting T-cell activity; however, treatment with RMC-7977 or RMC-7977 + palbociclib did not lead to changes in PD-1 surface levels, nor in the surface levels of LAG-3, another marker of T-cell exhaustion (Supplementary Fig. S4Q and S4R; ref. 48). These results contrast with our observations with MEK and CDK4/6 inhibition, which resulted in a marked increase in T-cell exhaustion (32). Therefore, as we did not observe treatment-induced increases in T-cell exhaustion and as the clinical efficacy of ICB treatment is limited in PDAC (49), we explored alternative mechanisms that could contribute to reduced tumor-cell surveillance by T cells.

One such mechanism with known relevance in PDAC involves defective interactions between T cells and professional antigen-presenting cells (APCs), such as DCs, which typically support helper CD4 T-cell and cytotoxic CD8 T-cell function by providing co-stimulatory signals (50–52). Notably, flow cytometry of tumor-infiltrating CD45 populations revealed a reduced abundance of DCs in tumors following RMC-7977 + palbociclib treatment compared with vehicle-treated controls (Fig. 3B; Supplementary Fig. S4N). More broadly, DCs and other APC populations demonstrated lower cell surface levels of CD40 in response to RMC-7977 and RMC-7977 + palbociclib treatment (Supplementary Fig. S4S). As CD40 is essential for APC–lymphocyte cross-talk, we reasoned that the observed lack of T-cell function might be due to reduced DC numbers and/or insufficient CD40 signaling by APCs.

### CD40 Agonism Dramatically Extends Responses to RMC-7977 + Palbociclib

CD40 agonists, which are currently in clinical trials for various cancer types, including PDAC, promote APC upregulation of co-stimulatory molecules and cytokines that are essential for T-cell activation and proliferation (53). Given the evidence of deficient CD40 signaling and the high degree of T-cell infiltration, we assessed the therapeutic potential of FGK4.5, a mouse monoclonal CD40 agonistic antibody (53), to rescue the observed lack of T-cell activity and stimulate an effective antitumor immune response.

**Figure 4.**
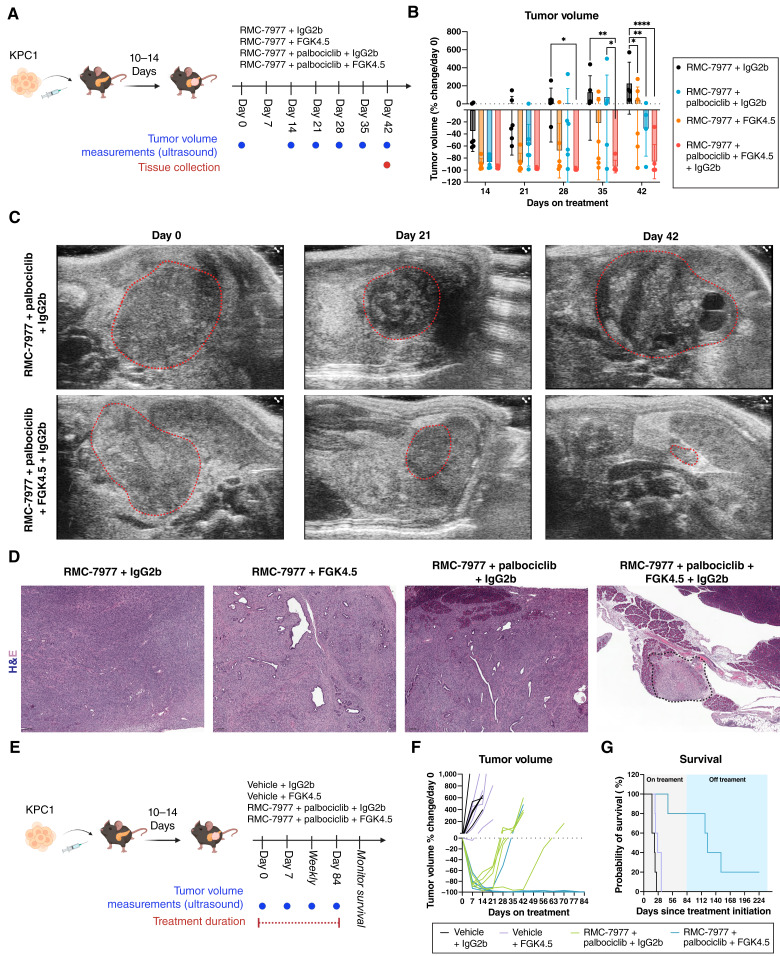
A triple combination consisting of RMC-7977, palbociclib, and a CD40 agonist maintains long-term tumor control despite the presence of residual disease. **A,** Scheme of experimental design (KPC1 orthotopic transplant into wild-type C57Bl/6 mice). **B,** Percentage change in tumor volume compared with day 0 as measured by weekly ultrasound. The *y*-axis indicates the percentage change in tumor volume, and the *x*-axis indicates the time point after treatment initiation. Each dot represents an individual mouse (*n* = 6 per treatment group at treatment initiation). Statistical testing: Two-way ANOVA with multiple comparisons (comparing each treatment group against every other treatment group within the same time point), with Tukey testing to correct for multiple comparisons. Only statistically significant comparisons are shown. At 28 days, *n* = 5 RMC-7977 + IgG mice remained, and *n* = 6 remained for all other treatment groups. At 35 days, *n* = 5 RMC-7977 + IgG mice and RMC-7977 + palbociclib + IgG mice remained, and *n* = 6 RMC-7977 + FGK4.5 mice and RMC-7977 + palbociclib + FGK4.5 mice remained. At 42 days, *n* = 4 RMC-7977 + IgG mice and RMC-7977 + palbociclib + IgG remained and *n* = 6 RMC-7977 + FGK4.5 mice and RMC-7977 + palbociclib + FGK4.5 mice remained. **C,** Representative snapshots of ultrasound imaging from RMC-7977 + palbociclib + IgG2b (top row) or RMC-7977 + palbociclib + FGK4.5 (bottom row) at indicated time points. Snapshots show the largest tumor cross-section. The red dashed line indicates tumor borders. **D,** Representative hematoxylin and eosin (H&E) images of residual tissue following 42 days of indicated treatment. The black dashed line surrounds residual tumor tissue. **E,** Scheme of experimental design (KPC1 orthotopic transplant into wild-type C57Bl/6 mice). **F,** Spaghetti plot showing percentage change in tumor volume compared with day 0 as measured by weekly ultrasound. The *y*-axis indicates the percentage change in tumor volume, and the *x*-axis indicates the time point after treatment initiation. Each line represents an individual mouse. **G,** Probability of survival (%) following treatment with indicated agents. RMC-7977 + palbociclib + IgG2b is not shown, as mice were euthanized prior to reaching humane endpoints. (**A** and **E,** Created with BioRender.com.)

In mice bearing orthotopic tumors derived from KPC1 or KPC2 cells, a triple combination consisting of RMC-7977, palbociclib, and FGK4.5 resulted in a more durable response compared with either RMC-7977 + FGK4.5 or RMC-7977 + palbociclib (Fig. 4A and B; Supplementary Fig. S5A and S5B). This effect appeared to be the result of prolonged control of residual disease, as initial regressions from RMC-7977 alone or in combination with palbociclib were only slightly enhanced by FGK4.5 (Fig. 4B; Supplementary Fig. S5B). Although RMC-7977 and FGK4.5 also showed combinatorial activity in tumors, this double combination was inferior to that of RMC-7977 + palbociclib + FGK4.5 in both KPC1 and KPC2 models (Fig. 4B; Supplementary Fig. S5B).

We also assessed the antitumor activity of RMC-7977 + palbociclib + FGK4.5 in mice bearing tumors derived from KPC4 cells, which produce more differentiated tumors with glandular structures and dense stroma (Supplementary Fig. S5C–S5F). In this model, monotherapy with RMC-7977 triggered tumor regression in only one of five mice 14 days after treatment initiation, while the combination of RMC-7977 + palbociclib, with or without the CD40 agonist FGK4.5, led to deep regressions at day 14 that persisted through at least 42 days of treatment (Supplementary Fig. S5E). Importantly, this translated to a significant survival extension in mice treated with RMC-7977 + palbociclib compared with RMC-7977 alone, which was further significantly extended in mice treated with the triple combination (Supplementary Fig. S5F). Assessing treatment-induced changes in histology in this KPC4 model revealed that acute treatment (7 days) with either RMC-7977 or RMC-7977 + palbociclib increased the density of differentiated ductal structures compared with vehicle, palbociclib, FGK4.5, or the triple combination of RMC-7977 + palbociclib + FGK4.5 (Supplementary Fig. S5G). Notably, in triple combination–treated tumors, a marked increase in acellular tissue was apparent (Supplementary Fig. S5G), and in line with this observation, the triple combination triggered an expansion of tissue area staining positive for the fibroblast marker podoplanin (Supplementary Fig. S5H and S5I), possibly indicative of a wound healing response or immune-driven stromal activation. Therefore, in mice bearing orthotopic tumors derived from three cell lines, we observed that treatment with the triple combination induced a prolonged state of stable disease.

### The RMC-7977 + Palbociclib + FGK4.5 Triple Combination Induces Stable Disease

In two of three models (KPC1 and KPC2), the triple combination led to undetectable tumors in a fraction of mice as indicated by ultrasound at 42 days after treatment initiation (Fig. 4B and C). To assess whether the triple combination completely eradicated disease, we isolated pancreatic tissues and assessed them histologically for evidence of residual tumor cells (Fig. 4D). Upon dissection, clearly visible masses were observed in tissues from the majority of mice treated with RMC-7977 alone, RMC-7977 + FGK4.5, or RMC-7977 + palbociclib, which were confirmed to be tumor tissue by hematoxylin and eosin staining (Fig. 4D). In contrast, no visible tumor masses were observed in pancreatic tissue from mice treated with RMC-7977 + palbociclib + FGK4.5 that showed near-complete tumor regressions by ultrasound; however, histological examination did reveal small clusters of residual tumor cells (Fig. 4B–D). This residual disease appeared stable, as mice treated with the triple combination showed few relapses even after 84 days of treatment (Fig. 4E and F). Nevertheless, these tumor cells were viable, as tumors relapsed in three out of four surviving mice following treatment withdrawal (Fig. 4G). Therefore, it appears that the triple combination triggers a state in which viable tumor cells persist but are controlled by a mechanism enhanced by CD40 agonism.

### CD4 T Cells Create a State of Equilibrium That Prevents Outgrowth of Residual Tumor Cells Following Treatment with Triple Combination

The prolonged maintenance of residual tumor cells via immune surveillance is reminiscent of tumor-immune “equilibrium,” a proposed phase of cancer immunoediting where transformed cells are not completely eradicated but remain controlled and held in balance by the immune system, resulting in long-term control (54–58). CD40 agonism can activate a range of immune cell types (53, 59) that could presumably enhance antitumor immunity and create a state of equilibrium, either alone or in combination.

To determine whether and how residual tumor cells might be kept in check by CD40 agonist-induced immune surveillance, we performed scRNA-seq on the CD45^+^ immune compartment of tumors treated with vehicle, palbociclib, RMC-7977, RMC-7977 + palbociclib, or RMC-7977 + palbociclib + FGK4.5 at both 7 and 14 days after treatment initiation (Supplementary Fig. S6A). Strikingly, the triple combination led to shifts in the fraction of each subpopulation; most notably, there was an expansion of T cells and a decrease in myeloid cells, as well as transcriptional changes consistent with a more immunostimulatory microenvironment (Supplementary Fig. S6B and S6C).

Though both CD4 and CD8 T cells appeared to expand dramatically, their inefficient capture in some treatment groups prevented comparison of treatment-induced changes in their states (Supplementary Fig. S6D and S6E). The transcriptional landscape of type 2 conventional DCs, which activate CD4 T cells, was markedly shifted in tumors from mice treated with RMC-7977, with RMC-7977 + palbociclib, and most notably with the triple combination (Supplementary Fig. S6F and S6G). Mature DCs enriched in immunoregulatory molecules, which may restrain antitumor immunity (60), also underwent a transcriptional shift in the triple combination–treated tumors, whereas type 1 conventional DCs were not detected following treatment with the triple combination (Supplementary Fig. S6F and S6G). The transcriptional landscape of triple combination–treated macrophages and monocytes shifted toward an immunostimulating monocyte state (Supplementary Fig. S6H and S6I), and though genes encoding the T-cell co-stimulatory molecules CD80 and CD86 were slightly diminished, the expression of *Cd83*, known to limit inflammation in macrophages, and that of *Cd40*, encoding the T cell–licensing receptor CD40, were elevated (Supplementary Fig. S6J). Importantly, macrophage and monocyte expression of the immune inhibitory marker genes *Mrc1* and *Arg1* were diminished (61) though no change in the proinflammatory marker *Il1b* was observed (Supplementary Fig. S6J). Further suggestive of a shift toward a monocyte state, *Ly6c2* was elevated in response to the triple combination (Supplementary Fig. S6J). B cells also acquired a phenotype consistent with an immunostimulatory role (Supplementary Fig. S6K and S6L), as indicated by an expansion of cells expressing the co-stimulatory molecules *Cd40* and *Cd86*, as well as *Cd69* and *Cxcr5*, which have been linked to T-cell homing and immunotherapy response (Supplementary Fig. S6M; ref. 62). Though the expression of the regulatory B-cell marker *Il10* was not observed, *Cd274*, encoding the immunosuppressive marker PD-L1, was elevated following 14 days of treatment with the triple combination (Supplementary Fig. S6M).

Across several immune cell subtypes, the triple combination appeared to upregulate the expression of genes involved in antigen presentation, most strikingly in B cells, macrophages, and DCs, suggestive of enhanced crosstalk with T cells (Supplementary Fig. S6N). As expected, Cd40 expression was observed on DCs, B cells, and myeloid cells (Supplementary Fig. S6O), implicating these cells as direct targets of the CD40 agonist FGK4.5.

**Figure 5.**
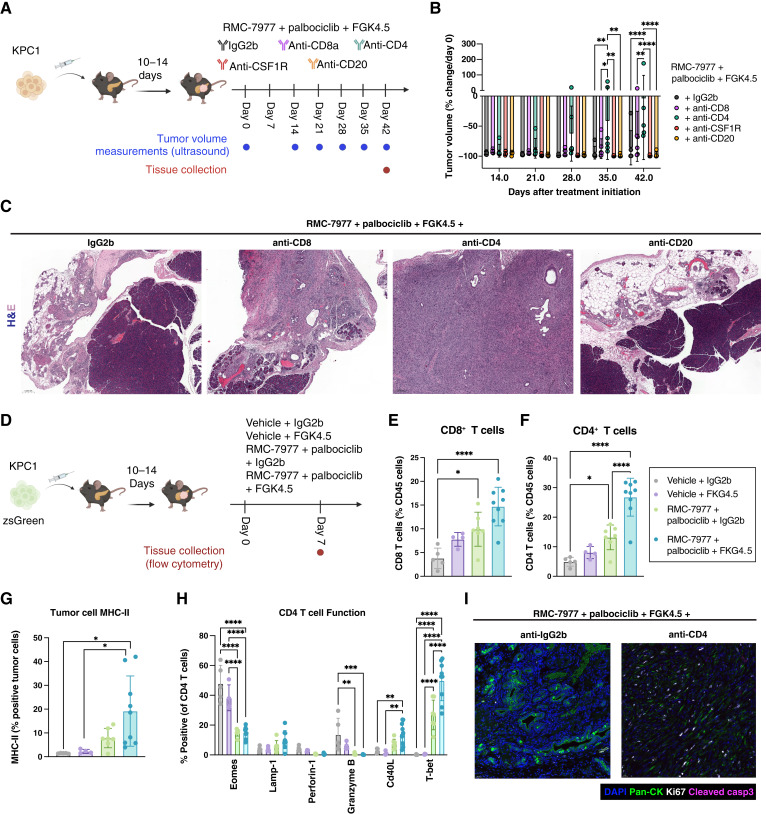
CD4 T cells prevent outgrowth of residual disease. **A,** Scheme of experimental design (KPC1 orthotopic transplant into wild-type C57Bl/6 mice). **B,** Percentage change in tumor volume compared with day 0, as measured by weekly ultrasound. The *y*-axis indicates the percentage change in tumor volume, and the *x*-axis indicates the time point after treatment initiation. Each dot represents an individual mouse. Statistical testing: Two-way ANOVA with multiple comparisons (within each time point, comparing treatment groups with every other treatment group) and correcting for multiple comparisons with a Tukey test. All statistically significant comparisons are shown. **C,** Representative hematoxylin and eosin (H&E) images of residual tissue following 42 days of indicated treatment. **D,** Scheme of experimental design for **E–H** (KPC1-ZsGreen orthotopic transplant into wild-type C57Bl/6 mice). **E,** Fraction of CD8^+^ T cells out of total CD45 cells by flow cytometry at 7 days after the initiation of indicated treatments. See **F** for the legend. Statistical testing for **E–G**: Ordinary one-way ANOVA with multiple comparisons, comparing the means of each treatment group with every other treatment group and correcting for multiple comparisons using a Sidak test. Only relevant statistically significant comparisons are shown. **F,** Fraction of CD4^+^ T cells out of total CD45 cells by flow cytometry at 7 days after the initiation of indicated treatments. Each dot represents an individual mouse. **G,** Fraction of tumor cells (%) positive for MHC-II by flow cytometry at 7 days after the initiation of indicated treatments. See **F** for the legend. Each dot represents an individual mouse. Statistical testing: Ordinary one-way ANOVA, comparing the means of every treatment group with every other treatment group and correcting for multiple comparisons with a Sidak test. All statistically significant comparisons are shown. **H,** Fraction of CD4 T cells (%) positive for indicated cytotoxicity and activation of Th1 markers by flow cytometry 7 days after treatment initiation. Statistical testing: Two-way ANOVA with multiple comparisons, comparing the mean of each treatment group with every other treatment group within the relevant time point and correcting for multiple comparisons with a Tukey test. Only statistically significant comparisons are shown. **I,** Representative IF images of residual tissue following 42 days of indicated treatments (see scheme in **A**) staining for pan-CK, Ki67, cleaved caspase 3, and DAPI. (**A** and **D,** Created with BioRender.com.)

In light of this marked remodeling of immune cells that may have a direct antitumoral role or stimulate the action of other effector immune cells, such as T cells, we next assessed the functional requirement of various immune cell subsets for the durability of the RMC-7977 + palbociclib + FGK4.5 response using depleting antibodies. To this end, tumor-bearing mice were treated with the triple combination and depleted of macrophages and monocytes (anti-CSF1R), CD8 T cells, CD4 T cells, and B cells (anti-CD20) or treated with an IgG2 isotype control (Fig. 5A). While neither macrophage/monocyte nor B-cell depletion impacted tumor responses to the triple combination, depletion of CD4 T cells and, to a lesser degree, CD8 T cells accelerated tumor relapse (Fig. 5B). Though we previously reported a role for CD8, but not CD4, T cells in the setting of an MEK inhibitor–based combination regimen with palbociclib and ICB (32), these results implicated CD4 T cells as crucial for immune surveillance provoked by the RAS(ON)-based triple combination, potentially uniquely poised to respond in this context because of the substantial tumor apoptosis triggered by RMC-7977, but not MEK inhibition (36, 37), which can result in the release of tumor antigens (63).

Indeed, additional characterization of tumors following treatment with the triple combination supported the notion that CD4 T cells were responsible for maintaining equilibrium. First, histological examination of tissues 42 days after treatment confirmed that while mice treated with the triple combination (and an IgG2b control) exhibited evidence of residual tumor cells, those treated with the triple combination and a CD4-depleting antibody showed clear tumor outgrowth (Fig. 5C). Second, analysis of tumor-infiltrating immune cells 7 days after treatment showed that both CD4 and CD8 T-cell fractions were elevated following RMC-7977 + palbociclib treatment, with a notably higher fraction of CD4 T cells in tumors treated with the triple combination (Fig. 5D–F). A triple combination–induced elevation in CD3 and CD4 T cells was also observed in the more differentiated KPC4 model, potentially enabled by the observed expansion of CD11c^+^ cells (Supplementary Fig. S7A–S7F). Apparently, the senescent-like state induced by RMC-7977 and palbociclib facilitates CD4 T cell–mediated surveillance of residual tumor cells upon the addition of the CD40 agonist.

### CD40 Engages CD4 T Cells to Maintain Tumors in Equilibrium

CD4 T cells have a broad range of functions that can both enhance and inhibit immune responses (64). One canonical role for CD4 T cells is to act as “helper” cells, of which there are several subsets, to coordinate immune responses. Notably, Th1 CD4 T cells aid in antitumor immunity by stimulating the effector functions of CD8 T cells and B cells (65). However, the limited role of B cells and CD8 T cells in triple combination mediated tumor control suggests that CD4 T cells independently contribute to tumor surveillance.

CD4 T cells recognize antigens presented by MHC-II, and while MHC-II expression is typically restricted to professional APCs and some subsets of CAFs (66), in settings of high IFN-γ, it can also be expressed by other cell types, including cancer cells (67). Strikingly, we found that the triple combination led to increased MHC-II on tumor cells 7 days after treatment initiation (Fig. 5G), indicative of possible direct cross-talk between residual tumor cells and CD4 T cells.

Although specialized subsets of CD4 T cells with cytotoxic activity against MHC-II expressing tumor cells have been linked to effective immunotherapy responses (68), CD4 T cells from tumors following triple combination treatment showed downregulation of markers of T-cell cytotoxicity, including perforin, granzyme B, and Eomes compared with those of vehicle-treated tumors by flow cytometry, while a high fraction of CD4 T cells were positive for the T-cell activation marker CD40L and the Th1-defining transcription factor T-bet (Fig. 5H). Thus, it is unlikely that an increase in direct CD4-mediated cytotoxicity is responsible for the sustained regressions that the triple combination produced.

Interestingly, while tumor equilibrium can arise through a delicate balance between the fraction of proliferating tumor cells and those that are killed by T cells (55), IF analysis of residual tumor tissue indicated that cytokeratin-positive tumor cells were neither proliferating nor undergoing apoptosis, as little-to-no staining for Ki67 and cleaved caspase 3 was observed (Fig. 5I). By contrast, ample evidence of proliferation was observed in tumor cells following CD4 T-cell depletion (Fig. 5I). These data raise the possibility that, in the setting of triple combination therapy, CD4 T cells can help sustain residual tumor cells in a senescent-like state.

### Th1 CD4 T Cells Coordinate an IFN-γ–Dependent Reinforcement of Senescence-Associated Proliferative Arrest

The capacity of CD4 T cells to reinforce tumor cytostasis parallels findings that Th1 CD4 T cells can induce senescence-like states in cancer cells through TNF-α and IFN-γ secretion (69). As we found that RMC-7977 plus palbociclib treatment led to a marked increase in T-bet–positive CD4 T cells, an effect further amplified by FGK4.5 co-administration (Fig. 5H), we explored whether a similar IFN-γ–dependent senescence induction might be at play.

**Figure 6.**
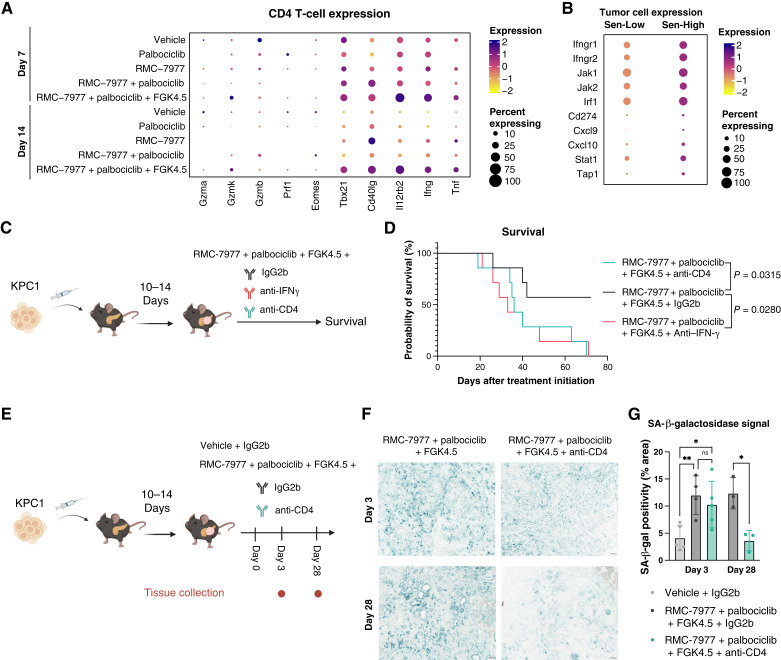
CD4 T-cell production of IFN-γ enables long-term tumor control. **A,** Expression of select cytotoxic and Th1-related genes in CD4 T cells from the scRNA-seq dataset. **B,** Expression of select genes encoding IFN-γ sensing machinery and IFN-γ–inducible genes in Sen-High versus Sen-Low cells. **C,** Scheme of experimental design (KPC1 orthotopic transplant into wild-type C57Bl/6 mice). **D,** Probability of survival (%) in mice following treatment with indicated agents. Statistical testing: A log-rank (Mantel–Cox) test was performed to compare survival curves, and *P* values are shown. **E,** Scheme of experimental design (KPC1 orthotopic transplant into wild-type C57Bl/6 mice). **F,** Representative images of pancreatic tumor tissues following detection of β-galactosidase activity using the chromogenic substrate X-Gal. **G,** Quantification of the fraction of SA-β-gal positive area out of total area (%). Each dot represents an individual mouse (*n* = 3–5 per treatment group), and the value shown is the average of 3 ∼159,000 µm^2^ regions per mouse tumor tissue (with the exception of two mice, where tumor tissue was only large enough to quantify 1–2 regions). Statistical testing: One-way ANOVA, comparing the mean of every column with the mean of every column within that time point and correcting for multiple comparisons with a Holm–Sidak test. All comparisons are shown. (**C** and **E,** Created with BioRender.com.)

Investigating the transcriptional state of CD4 T cells that expand in tumors following treatment with the triple combination confirmed that these CD4 T cells expressed high levels of Th1 markers *Tbx21* and *Il12rb1*, but not genes encoding cytotoxic effectors such as granzymes (*Gzma*, *Gzmk*, *Gzmb*), perforin (*Prf1*), or the transcription factor Eomes (Fig. 6A). Notably, CD4 T cells from mice treated with the triple combination demonstrated a pronounced upregulation in the expression of *Ifng*. We also observed that tumor cells that engaged senescence-related transcriptional programs, or “Sen-High” cells (Fig. 2D), upregulated IFN-γ signaling machinery, including *Ifngr1*, *Ifngr2*, *Jak1*, *Jak2*, *Irf1*, and *Stat1*, as well as IFN-γ target genes *Cxcl9*, *Cxcl10*, and *Tap1* (Fig. 6B). These results are in line with a previously reported enhanced IFN-γ sensing capacity of senescent cells (31) and suggest a positive, self-amplifying feedback loop that may drive further CD4 T-cell expansion. Functionally, IFN-γ contributes to the maintenance of tumor cells in a state of equilibrium, as antibody-mediated neutralization of IFN-γ phenocopied CD4 T-cell depletion, accelerating tumor escape in triple combination–treated mice (Fig. 6C and D).

Further supportive of the notion that CD4 T cells sustain cancer cells in a senescent-like state, tumors from mice treated with the triple combination exhibited persistent SA-β-galactosidase positivity through 28 days of treatment, which was diminished upon CD4 T-cell depletion (Fig. 6E–G).

These results are consistent with a model whereby a triple combination consisting of RMC-7977, palbociclib, and a CD40 agonist engages Th1 CD4 T cells to reinforce the senescence-associated proliferative arrest driven by combined RMC-7977 and palbociclib, resulting in stable equilibrium. Our study demonstrates that agents that combine with RAS inhibition, as shown here by the addition of palbociclib, can shift persister tumor cells into a senescent-like state that can potentiate immune-mediated control of residual tumor cells.

## Discussion

Given the high prevalence of *KRAS* mutations in PDAC, the recent discovery of small molecule RAS inhibitors holds promise for transforming the treatment landscape. However, acquired resistance to RAS inhibitors occurs in preclinical and clinical settings (8, 10, 15–17, 20), highlighting the importance of identifying effective combination strategies. Our study suggests that reprogramming persistent tumor cells that survive RAS inhibition into a senescent-like state can prolong responses and provide an opportunity to engage antitumor immunity. In agreement, a recent report using RAS and CDK4/6 inhibitors in cell lines and organoids also notes a prominent induction of senescence with the drug combination (bioRxiv 2025.01.11.632518). In immune-competent PDAC models, the acquisition of a senescent-like state appears to trigger T-cell infiltration into the tumor. While this alone is insufficient to engage antitumor immunity, incorporating CD40 agonist treatment leads to durable, immune-dependent responses. Long-term tumor control driven by this triple combination involves CD4 T cells promoting a state of equilibrium that maintains residual tumor cells in a nonproliferative state.

Cellular senescence, initially described as an RB- and p53-dependent process associated with the replicative exhaustion of fibroblasts (70–73), can be induced in tumor cells following various cancer therapies (26). Although canonical senescence regulators are often disabled during tumorigenesis, therapy-induced senescent states can still produce proinflammatory environments that either support cancer progression or promote immune surveillance depending on the context (29). While we noted an infiltration of T cells into tumors harboring tumor cells in a senescent-like state, DCs were depleted and showed diminished surface levels of CD40, indicating an insufficiency in signals needed to activate effector T cells. CD40 agonism is known to activate the CD40 receptor on APCs and, in doing so, stimulate antitumor T-cell responses. In line with this, the addition of a CD40 agonist to the RMC-7977 + palbociclib combination produced more durable treatment responses, potentially by rescuing this deficiency. These findings may also be relevant in the setting of mutant selective RAS inhibition, as therapy-induced reductions in DCs have also been observed in a preclinical model with a KRAS^G12D^ selective inhibitor (17).

Our observations contribute to the understanding of the heterogeneity in biological and molecular mechanisms associated with therapy-induced senescence. Prior work shows that senescent cells become sensitized to environmental IFN-γ and upregulate antigen-presenting machinery, enhancing their immunogenicity (31). However, depending on the context, senescence-promoting therapies can provoke various types of immune responses, including NK cell engagement, altered macrophage function, and CD4- and CD8-mediated T-cell surveillance (31, 32, 35, 44). Likewise, RAS pathway inhibition can produce diverse immunological responses, such as sensitizing lung cancer models to anti–PD-1 therapy or PDAC to direct CD8 T cell–mediated attack (45, 74). These differences may involve variability in tissue context (32, 35), intrinsic differences in the immunogenicity of transplanted cell lines (75), the immunogenicity of MHC-I or MHC-II neoepitopes present (76, 77), or a lack of neoepitopes (78). Alternatively, for RAS(ON) multi-selective inhibitors such as RMC-7977, the effects of wild-type RAS inhibition on normal immune cells may also play a role (79). Evaluation of on-treatment biopsies will be necessary to establish the nature and heterogeneity of treatment-induced immune responses in patients.

In our PDAC models, the sustained tumor control imparted by the triple combination therapy is reminiscent of the phenomenon of “tumor equilibrium,” first described by Schreiber and colleagues as a state where tumor outgrowth is held in balance by the immune system (80). Equilibrium was initially envisioned to involve continuous immune surveillance of proliferating tumor cells and is supported by experimental evidence of dormant tumors arising in immunocompetent mice that escape upon immunosuppression, as well as clinical observations of tumor outgrowth in immune-compromised patients receiving organs from former cancer patients (55, 81, 82). In this study, the addition of CD40 agonism to the RMC-7977 + palbociclib combination maintained tumor cells in a CD4 T cell–mediated state of cytostasis, seemingly outside their canonical “helper” role and not through direct cytotoxicity, but instead consistent with their described ability to drive or reinforce the senescent state (69, 83). To our knowledge, our study represents the first report of the induction of tumor-immune equilibrium induced by targeted therapies in combination with immunotherapies and implies that this state can be a beneficial outcome of treatment.

Other studies demonstrating combinatorial activity between immunotherapies and RAS inhibitors beg the question of in which contexts various immunotherapy strategies might be most effective (74, 84, 85). Checkpoint blockade may be most effective in tumors with high PD-L1 expression (86) or those showing increased evidence of T-cell exhaustion following KRAS inhibition, as these phenotypes suggest an ongoing although inadequate, immune response and overactivation of immune checkpoint pathways. In settings where DCs are diminished or where defective T-cell priming is observed, CD40 agonists, which have shown promising clinical activity in combination with chemotherapy (87), could help restore effective immune activation by enhancing antigen presentation and co-stimulation. Additionally, since mRNA vaccines showed early success in PDAC patients in the neoadjuvant setting, they may also be effective in settings of low residual disease following tumor “debulking” with RAS inhibitors (25). TLSs are known positive prognostic indicators of response to immunotherapy (88, 89), and correspondingly, in our studies, one model in which TLSs emerged showed more durable responses than one without. While correlative, our findings highlight the need to further explore TLSs as a predictive biomarker in RAS inhibitor combinations, potentially guiding optimal immunotherapy use.

In summary, our preclinical study illustrates how inducing senescent-like states in PDAC cells that survive RAS inhibition can prolong tumor responses and prime the residual tumor mass to the effects of immunotherapy. In doing so, we reveal the previously unappreciated ability of the senescence program to engage tumor-immune equilibrium and highlight the importance of CD4 T cells in antitumor immunity potentiated by RAS inhibition. These findings prompt additional studies exploring the mechanisms by which CD4 T cells maintain equilibrium and contribute to immunotherapy responses in PDAC and may reveal novel immunotherapy strategies that leverage the ability of CD4 T cells to maintain, or possibly eliminate, residual disease. Such strategies hold potential for tumor types, such as pancreatic cancer, which are considered “cold” or relatively refractory to immune modulation, to benefit from immunotherapy.

## Methods

### Cell Culture and Derivation

Murine KPC1, KPC2, and KPC4 PDAC cell lines were generated as previously described (32). In brief, tumors arising in *Pdx1-Cre*; *LSL-Kras*^*G12D/+*^, and *LSL-Trp53*^*R172H/+*^ mice were minced with razor blades and then dissociated in 1 mg/mL collagenase type V (C9263; Sigma-Aldrich) and dispase (17105041, Gibco) diluted in Hank’s Balanced Salt Solution (HBSS; 14025-076; Life Technologies) at 37°C with mild agitation for up to 1 hour. The cell suspension was then plated on 10 cm^2^ culture dishes coated with 100 mg/mL collagen (PureCol, 5005; Advanced BioMatrix) and grown in complete DMEM containing 10% FBS and 100 IU/mL penicillin/streptomycin. Primary cultures were passaged at least three times to remove fibroblast contamination. KP-4 cells (JCRB0182, RRID:CVCL_1338) for xenograft tumor inoculation were grown in Iscove's modified Dulbecco’s medium supplemented with 20% heat-inactivated FBS and 100 U/mL penicillin and 100 μg/mL streptomycin. The cells growing in the exponential growth phase were harvested and counted for tumor inoculation. Cells were maintained in a humidified incubator at 37°C with 5% CO_2_. All cells used tested negative for mycoplasma.

### Animal Studies

All animal experiments in this study were performed in accordance with protocols approved by the Memorial Sloan Kettering Institutional Animal Care and Use Committee (approval number: 11-06-018). The mice were housed with a 12-hour light/dark cycle between 8:00 am and 8:00 pm in a temperature-controlled room (22 ± 1°C) with free access to water and food.

### “KPC” GEMM PDAC Model

*Pdx1-Cre* (RRID:IMSR_JAX:014647), *LSL-Kras*^*G12D/+*^ (RRID:IMSR_JAX:008179), and *Trp53*^*fl/fl*^ (RRID:IMSR_JAX:008462) strains on a C57Bl/6 background were interbred to obtain *Pdx1-Cre, LSL-Kras*^*G12D/+*^, and *Trp53*^fl/+^ (KPC) mice. Both male and female KPC mice ranging from 2.5 to 12 months of age were used for treatment studies upon confirmation of tumor development. Mice were monitored for tumor development by palpation and ultrasound using a Vevo 2100 (RRID:SCR_015816) and were enrolled and randomized into treatment groups once tumors reached 35 to 350 mm^3^. Ultrasound imaging was repeated weekly during treatment to assess changes in pancreatic tumor burden.

### Orthotopic PDAC Cell Line Transplant Model

To rapidly generate synchronized cohorts of mice bearing PDAC tumors, KPC1 or KPC2 PDAC cells (or variants of these lines, e.g., KPC1-ZsGreen, as indicated in schemes) were orthotopically transplanted into the pancreases of 6- to 10-week-old C57Bl/6N (RRID:MGI:2159965) or 7- to 9-week-old NSG female mice (RRID:BCBC_1262). For KPC1 cells, 2.5 × 10^4^ cells were transplanted into each mouse. For KPC2 cells, 1 × 10^5^ cells were transplanted into each mouse. For KPC4 cells, 3 × 10^4^ cells were transplanted into each mouse. Cells were resuspended in a 1:1 serum-free DMEM/Matrigel (Corning, 356231) mix of 25 μL volume and injected into the exposed pancreata of each anesthetized mouse using a Hamilton syringe fitted with a 26-gauge needle. Mice were monitored for tumor development (10–14 days after transplantation for KPC1 cells, 21 days for KPC2 and KPC4 cells) by palpation and ultrasound and then assigned by stratified randomization (by baseline tumor volume) into treatment groups.

### Animal Studies Using Xenograft Tumor Models

Studies were conducted at the contract research organizations (KP-4 cell line-derived xenograft (CDX) at WuXi AppTec, all patient-derived xenograft (PDX) at GenenDesign). All CDX/PDX mouse studies and procedures related to animal handling, care, and treatment complied with all applicable regulations and guidelines of the Institutional Animal Care and Use Committee at each facility with their approvals. Female BALB/c nude mice 6 to 12 weeks old were used. Animal vendors include Beijing Vital River/VR Laboratory Animal Co. Ltd. and Shanghai Sino-British SIPPR/BK Laboratory Animal Co. Ltd.

To generate subcutaneous KP-4 CDX, 5 × 10^6^ KP-4 cells in the exponential phase of growth were harvested for tumor cell inoculation, and each mouse was inoculated in the right flank with tumor cells. Treatments were initiated when the average tumor volume reached 130 to 200 mm^3^ for tumor growth evaluation. Tumor diameter was measured in two dimensions using a digital caliper, and the tumor volume in mm^3^ was calculated using the following formula: Volume = ((width)^2^ × length)/2). Tumors were measured twice weekly.

The human primary cancer PDX models were generated using fresh tumor fragments obtained from the hospital with written informed consent from patients in accordance with protocols approved by the hospital’s institutional ethics committee. Tumor fragments were subcutaneously serially passaged in immunodeficient mice and cryopreserved for further use. Recovered tumor fragments were implanted into the right flanks of immunodeficient mice; treatment was started when the average tumor volume reached 150 to 350 mm^3^.

The percentage mean tumor volume change from baseline was graphed in the waterfall plots. The modified Response Evaluation Criteria in Solid Tumors (mRECIST) score was determined based on the percentage of mean tumor volume change, where mCR (complete response according to mRECIST) indicates greater than 80% regression, mPR (partial response according to mRECIST) indicates regression between 30% and 80%, mSD (stable disease according to mRECIST) indicates between 30% growth and 30% regression, and mPD (progressive disease accordng to mRECIST) indicates greater than 30% growth. Progression is defined as tumor volume doubling from baseline and is represented with Kaplan–Meier plots. A log-rank test with Benjamini–Hochberg multiple comparisons adjustment was used to compare treatment groups.

### Ultrasound Tumor Measurements (Orthotopic PDAC Models)

High-contrast ultrasound imaging was performed on a Vevo 2100 System with an MS250 13- to 24-MHz scanhead (RRID:SCR_015816) to stage and quantify pancreatic tumor burden. For KPC GEMMs, three-dimensional volumetric measurements were obtained using Vevo LAB software by sequentially measuring the circumference of tumor cross-sections from end to end in 5 to 15 parallel planes (parallel segmentation) through the 3D images to create a 3D volume. For orthotopic PDAC cell line transplant models, ultrasound measurements were performed at baseline and weekly thereafter unless otherwise indicated in experimental schemes, and tumor volume was estimated using two measurements: the diameter of the largest cross-section of the tumor (D1) and the diameter perpendicular to the largest cross-section (D2). Approximate tumor volume (mm^3^) was calculated using the following equation: (D1/2) × (D2/2) × ((D1 × D2)/2) × 4/3 × π.

### Sample Collection and Preparation for Histology, Flow Cytometry, and scRNA-Seq Experiments

For IHC and flow cytometric analysis or sorting of tumor cells for scRNA-seq studies, KPC1 PDAC cells were transduced with a pMSCV-IRES-GFP (Addgene, plasmid #20672; http://n2t.net/addgene:20672; RRID:Addgene_20672) or pMSCV-IRES-ZsGreen (cloned from pMSCV-IRES-GFP) retroviral expression vectors and ZsGreen or GFP positive cells were sorted using a Sony MA900 Multi-Application Cell Sorter (RRID:SCR_026300). KPC1-ZsGreen or KPC1-GFP cells were orthotopically transplanted into the pancreases of 6- to 10-week-old C57Bl/6N female mice. Mice were treated with indicated therapies for indicated amounts of time. Upon sacrifice, pancreas tumor tissue was dissected, and normal tissue was carefully trimmed away. Tumor tissue was split into pieces for downstream processing as follows: (i) Tissue was fixed in 10% formalin for 24 hours and then stored in 70% ethanol until paraffin embedding; (ii) tissue was fixed in 4% paraformaldehyde (PFA) in PBS for 24 hours, followed by cryoprotection in 30% sucrose for 4 hours, and then embedded in Tissue-Tek Optimal Cutting Temperature (OCT.) compound on dry ice; or (iii) tissue was digested in 1 mg/mL collagenase type V and 1 mg/mL DNase (Sigma, Dn25) in HBSS using a Miltenyi gentleMACS Octo Dissociator with Heaters Tissue Dissociator (RRID:SCR_020271) to obtain single-cell suspensions for flow cytometry or FACS.

### Small-Molecule RAS and CDK4/6 Inhibitor Treatments (*In Vivo*)

RMC-7977 (obtained from Revolution Medicines) was administered 3 times per week (e.g., Monday, Wednesday, and Friday) at 25 mg/kg by oral gavage. Palbociclib (LC Laboratories, P-7744) was administered 3 times per week (e.g., Monday, Wednesday, and Friday) at 100 mg/kg by oral gavage.

All dosing solutions for *in vivo* studies were formulated by the antitumor assessment core as follows: RMC-7977 was diluted in a 10:20:10:60 (%v/v/v/v) solution of DMSO/PEG400/Solutol/sterile water at 2.5 mg/mL. The same vehicle was used for all control groups. Palbociclib was diluted in 50 mmol/L sodium lactate (pH 4.0) at 10 mg/mL

### Depleting, Antagonistic, or Agonistic Antibody Treatments (*In Vivo*)

The depleting anti-mouse CD4 antibody (Bio X Cell, clone: GK1.5, RRID: AB_1107636) was administered 2 times per week at 200 µg/mouse (i.p.). The depleting anti-mouse CD8a antibody (Bio X Cell, clone: 2.43, RRID: AB_1125541) was administered 2 times per week at 200 µg/mouse (i.p.). The depleting anti-mouse CSF1R antibody (Bio X Cell, clone: AFS98, RRID: AB_2687699) was administered 2 times per week at 400 µg/mouse (i.p.). The depleting anti-mouse CD20 antibody (BioLegend cat. # 152104, clone: SA271G2, RRID:AB_262961) was administered 1 time per month at 250 µg/mouse (i.v.). The neutralizing anti-mouse IFN-γ antibody (Bio X Cell, clone XMG2.2, RRID: AB_1107694) was administered 2 times per week at 200 µg/mouse (i.p.). The agonistic anti-mouse CD40 antibody (Bio X Cell, clone: FGK4.5, RRID: AB_1107601) was administered every 14 days at 100 µg/mouse (i.p.).

### Flow Cytometry and FACS

#### Sample Preparation for Sorting of or Flow Cytometric Analysis of Tumor and Immune Cell Populations (*In Vivo*)

Immunophenotyping analysis of tumor and immune cell frequencies and states following treatments was performed by digesting tumors established by orthotopic transplant of KPC1-ZsGreen or KPC1-ZsGreen-Watermelon cells into C57Bl/6 mice. Pancreatic tumors were dissected from mice, and normal pancreas and splenic tissue were trimmed away. Tumors were placed into Miltenyi gentleMACS C Tubes containing 1.5 mL of a solution of 1 mg/mL collagenase V (Sigma-Aldrich, C5138) and 0.1 mg/mL DNase I (Sigma-Aldrich, DN25) in HBSS. Tumors were chopped with scissors into small pieces (1–2 mm), and an additional 3 mL of collagenase V/DNase solution was added. Tumors were incubated at 37°C on a gentleMACS Dissociator using the mTDK1 program. Tubes were then centrifuged, and pellets were resuspended in 2 mL of ACK Lysing Buffer (Quality Biological, 118-156-101) and incubated at room temperature for 5 minutes to lyse red blood cells and then quenched with PBS. Single-cell suspensions were then transferred through a 100 μm nylon cell strainer (Falcon, 352360).

#### Staining of Single-Cell Suspensions for Sorting or Flow Cytometric Analysis of Tumor and Immune Cell Populations (*In Vivo*)

Cells were washed, pelleted, and resuspended in the Molecular Probes LIVE/DEAD Fixable Blue Dead Cell Stain Kit (Thermo Fisher Scientific, L23105) at 1:500 in PBS or the Molecular Probes LIVE/DEAD Fixable Near-IR Dead Cell Stain Kit (Thermo Fisher Scientific, L10119) at 1:1,000 in PBS for 30 minutes at 4°C. Cell suspensions were then washed in PBS and stained in a cocktail of extracellular antibodies diluted in FACS buffer (2% FCS in PBS; see Flow Cytometry Antibodies) for 30 minutes at 4°C. At this point, cells were washed and resuspended in FACS buffer and sorted using the Sony MA900 to collect samples for scRNA-seq.

For staining of intracellular markers and subsequent analysis of tumor and immune cell populations by flow cytometry, cells were fixed and permeabilized using the Foxp3/Transcription Factor Staining Buffer Set (eBioscience, 00-5523-00) per user instructions. Cells were stained in a cocktail of intracellular antibodies (see Flow Cytometry Antibodies) diluted in permeabilization buffer for 30 minutes to overnight at 4°C. Finally, cells were washed and resuspended in FACS buffer and filtered into 5 mL Polystyrene Round-Bottom Tubes with Cell-Strainer Caps (Falcon, 352235). Flow cytometric data were acquired on a Cytek Aurora Spectral Analyzer (RRID:SCR_019826) or a Sony MA900. Single-stain controls were prepared by staining splenocytes or UltraComp eBeads Compensation Beads (Thermo Fisher Scientific, 01-2222-41) with the antibodies of interest or using cultured KPC1-ZsGreen or KPC1-GFP cells.

#### Analysis of Flow Cytometric Data

Data were analyzed using FlowJo (RRID:SCR_008520)—see gating strategies (Supplementary Fig. S8A and S8B). CD45^+^ cells were defined as LIVE/DEAD Blue negative, single cells staining positive for CD45. Conventional NK cells were defined by CD45^+^ cells staining positive for NK1.1 and negative for TCRαβ or TCRγδ. NKT-TCRαβ cells were defined as NK1.1^+^ cells staining positive for TCRαβ. NKT-TCRγδ cells were defined as NK1.1^+^ cells staining positive for TCRγδ. TCRαβ T cells were defined as CD45^+^NK1.1^−^ cells staining positive for CD3 and TCRαβ. CD8 T cells were defined by TCRαβ^+^ T cells staining positive for CD8 and negative for CD4. CD4 T cells were defined by TCRαβ^+^ T cells staining positive for CD4 and negative for CD8. Macrophages were defined by CD45^+^NK1.1^−^TCRαβ^−^F4/80^+^ cells. M1 macrophages were defined by macrophages staining positive for CD206. M2 macrophages were defined by macrophages staining negative for CD206. DCs were defined by CD45^+^NK1.1^−^TCRαβ^−^F4/80^−^CD11c^+^MHC-II^+^ cells. Neutrophils were defined by CD45^+^NK1.1^−^TCRαβ^−^F4/80^−^CD11c^−^CD11b^+^Ly6g(high)Ly6c(low). Monocytes were defined by CD45^+^NK1.1^−^TCRαβ^−^F4/80^−^CD11c^−^CD11b^+^Ly6g(mid)Ly6c(high). B cells were defined by CD45^+^NK1.1^−^TCRαβ^−^F4/80^−^CD11c^−^CD11b^−^MHC-II^+^.

#### Flow Cytometry Antibodies

The following extracellular antibodies were used for immunophenotyping analysis of mouse tumor tissue: BUV563 anti-mouse CD45 (clone: 30-F11, BD Biosciences, cat. # 612924, RRID:AB_2870209), PE/Dazzle 594 anti-mouse TCRβ chain (clone: H57-597, BioLegend, cat. # 109240, RRID:AB_2565655), BUV805 anti-mouse CD8 (clone: 53-6.7, BD Biosciences, cat. # 612898, RRID:AB_2870186), BUV661 anti-mouse CD4 (clone: GK1.5, BD Biosciences, cat. # 612974, RRID:AB_2870246), BV785 anti-mouse PD-1 (CD279; clone: 29F.1A12, BioLegend, cat. # 135225, RRID:AB_2563680), BV650 anti-mouse/anti-human CD44 (clone: IM7, BioLegend, cat. # 103049, RRID:AB_2562600), BUV737 anti-mouse CD69 (clone: H1.2F3, BD Biosciences, cat. # 612793, RRID:AB_2870120), PE/Cy7 anti-mouse CD223 (LAG-3; clone: eBioC9B7W, Invitrogen, cat. # 25-2231-82, RRID:AB_494214), BV711 anti-mouse TIM3 (clone: RMT3, BioLegend, cat. # 119727, RRID:AB_2716208), APC-Cy7 anti-mouse CD62L (clone: MEL-14, BioLegend, cat. # 104428, RRID:AB_830799), BUV395 anti-mouse γδ TCR (clone: GL3, BD Biosciences, cat. # 744118, RRID:AB_2742008), BV605 anti-mouse NK1.1 (clone: PK136, BioLegend, cat. # 108739, RRID:AB_2562273), BV421 anti-mouse CD25 (clone: PC61, BioLegend, cat. # 102033, RRID:AB_10895908), APC anti-mouse CD178 (FasL; clone: MFL3, BioLegend, cat. # 106609, RRID:AB_2813951), BV510 anti-mouse CXCR3 (clone: CXCR3-173, BioLegend, cat. # 126527, RRID:AB_2562204), PE anti-mouse CD186 (CXCR6; clone: SA051D1, BioLegend, cat. # 151103, RRID:AB_2566545), BUV395 anti-mouse CD11b (clone: M1/70, BD Biosciences, cat. # 563553, RRID:AB_2738276), BV785 anti-mouse CD11c (clone: N418, BioLegend, cat. # 117335, RRID:AB_11219204), APC anti-mouse F4/80 (clone: BM8, BioLegend, cat. # 123116, RRID:AB_893481), BV605 anti-mouse LY-6G (clone: 1A8, BD Biosciences, cat. # 563005, RRID:AB_2737946), BV711 anti-mouse Ly-6C (clone: HK1.4, BioLegend, cat. # 128037, RRID:AB_2562630), APC-Cy7 anti-mouse I-A/I-E (MHC-II; clone: M5/114.15.2, BioLegend, cat. # 107627, RRID:AB_1659252), BV650 anti-mouse CD206 (clone: C068C2, BioLegend, cat. # 141723, RRID:AB_2562445), AF700 anti-mouse CD86 (clone: PO3, BioLegend, cat. # 105122, RRID:AB_493723), PerCP/Cy5.5 anti-mouse Siglec-F (clone: E50-2440, BD Biosciences, cat. # 565526, RRID:AB_2739281), BV421 anti-mouse CD80 (clone: 16-10A1, BioLegend, cat. # 104725, RRID:AB_10900989), PE/Dazzle 594 anti-mouse CD40 (clone: 3/23, BioLegend, cat. # 124630, RRID:AB_2572185), PE-Cy7 anti-mouse PD-L1 (CD274; clone: 10F.9G2, BioLegend, cat. # 124314, RRID:AB_10643573), BUV737 anti-mouse CD19 (clone: 1D3, Thermo Fisher Scientific, cat. # 367-0193-82, RRID:AB_2895945), PE anti-mouse TCRβ chain (clone: H57-597, BD Biosciences, cat. # 553172, RRID:AB_394684), PE-Cy5 anti-mouse NK1.1 (clone: PK136, BioLegend, cat. # 108715, RRID:AB_493591), Pacific Blue anti-mouse B220 (CD45R; clone: RA.3-6B2, BioLegend, cat. # 103227, RRID:AB_492876), BV605 anti-mouse KLRG1 (MAFA; clone:2F1/KLRG1, BioLegend, cat. # 138419, RRID:AB_2563357), BV786 rat anti-mouse CD107a (LAMP-1; clone: 1D4B, BD Biosciences, cat. # 564349, RRID:AB_2738762), and BUV737 hamster anti-mouse CD154 (CD40L; clone: MR1, BD Biosciences, cat. # 741735, RRID:AB_2871105).

The following intracellular antibodies were used for immunophenotyping analysis of mouse tumor tissue: BUV496 anti-mouse CD3 (clone: 17A2, BD Biosciences, cat. # 741117, RRID:AB_2870707), AF532 anti-mouse Foxp3 (clone: FJK-16s, Thermo Fisher Scientific, cat. # 58-5773-82, RRID:AB_11218870), AF700 anti-mouse Ki67 (clone: B56, BD Biosciences, cat. # 561277, RRID:AB_10611571), Pacific Blue anti-mouse granzyme B (clone: GB11, BioLegend, cat. # 515408, RRID:AB_2562196), PerCP/Cy5.5 anti-T-bet (clone: 4B10, BioLegend, cat. # 644805, RRID:AB_1595593), PE anti-mouse EOMES (clone: Dan11mag, Thermo Fisher Scientific, cat. # 12-4875-80, RRID:AB_1603278), PE-Dazzle594 anti-mouse perforin (clone: S16009A, BioLegend, cat. # 154315, RRID:AB_2922482), and BV421 anti–T-bet (clone 4B10, BioLegend, cat. # 644815, RRID:AB_10896427).

### Functional Evaluation of Tumor-Cell Proliferative History

To track the proliferative history of tumor cells in response to various treatments, KPC1-ZsGreen PDAC cells were transduced with the “Watermelon backbone” vector (Addgene, plasmid #155258; http://n2t.net/addgene:155258; RRID:Addgene_155258). Tumor cells were cultured with doxycycline (1 µg/mL) to induce H2B-mCherry expression and then sorted on a SONY MA900 to select for H2B-mCherry–positive cells. KPC1-ZsGreen–Watermelon cells were orthotopically injected into NSG or Rosa26-CAGs-rtTA3 mice (JAX Strain #029627). Recipient mice were fed a doxycycline hyclate diet (200 ppm, Envigo, TD.180625) 2 days before surgery and maintained on doxycycline chow until 14 days after transplant (or longer where indicated), at which point tumor volume was measured by small animal ultrasound and mice were assigned by stratified randomization into treatment groups. Upon treatment initiation, mice were switched to doxycycline-free chow or maintained on doxycycline for an additional 7 days, as indicated in the experimental schemes. Following 7 days of treatment, mice were euthanized, and tumor tissue was collected and digested to obtain single-cell suspensions [see “Flow Cytometric Analysis of Tumor and Immune Cell Populations (*In Vivo*)”].

### scRNA-Seq Experiments

To investigate transcriptional changes in tumors in response to RMC-7977 monotherapy and combination regimens, scRNA-seq was performed. Cohorts of C57Bl/6 mice bearing orthotopic KPC1-zsGreen–derived tumors were generated (see “Orthotopic PDAC Cell Line Transplant Model”). Mice were treated as indicated in experimental schemes and euthanized at indicated time points. Upon euthanasia, pancreatic tumors were dissected and digested to obtain single-cell suspensions (see “Flow Cytometry and FACS”), which were FACS-sorted for CD45^−^, ZsGreen^+^ (tumor) cells. Tumor or CD45^+^ cells from two to three mice treated with the same treatment group were pooled into a single sample (see Supplementary Table S1), and cells were encapsulated and processed following the 10x Genomics user manual (Reagent Kit 5′ version 2).

Count matrices were generated by aligning sequencing reads using Cell Ranger (version 8.0.1; ref. 90) with default settings and the mouse reference transcriptome, GRCm39-2024-A. To remove potential ambient RNA contamination, CellBender’s “removebackground” function was applied (version 1.0; ref. 91). The resulting ambient-corrected count matrices were then analyzed using Seurat (version 5.1.0; ref. 92). Quality control was performed for each sample with custom thresholds, selected based on the distribution of quality control metrics. Key metrics included the percentage of mitochondrial and ribosomal reads, the number of features detected, and the per-cell library size. Since neutrophils exhibited overall lower library sizes compared with other cell types, quality control filters were adjusted accordingly.

For downstream analysis, Seurat’s standard procedures for normalization, dimensionality reduction, and clustering were followed. The number of principal components was determined using the Talus plot method (93). Cell counts were normalized using SCTransform, with cell cycle scores regressed out using the gene list from Kowalczyk and colleagues (94), along with the percentage of mitochondrial and ribosomal reads. Optimal clustering resolution was selected using the clustree R package (version 0.5.0; ref. 95), and clusters were assigned cell types based on the expression of known marker genes from the literature. Cluster identities were further validated by comparing them with annotations from ImmGen (96) using SingleR (version 2.6.0; ref. 97). This clustering approach was applied to each sample independently, allowing for the removal of sample-specific artifacts.

For immune cell analysis, batch effects were addressed by integrating cells across all samples using the fastMNN algorithm (98). To refine the cell population structure, subclustering was performed independently for major cell types (tumor cells, lymphoid cells, myeloid cells, vasculature, and fibroblasts). This approach enabled the identification of finer cell population structures and facilitated the removal of doublets and low-quality cells through inspection of population marker gene expression and quality control metrics.

Clusters were scored for the Mouse Hallmark Gene Set signatures of the Molecular Signatures Database using Seurat’s AddModuleScore function in combination with the msigdbr R package (R package version 2023.1.1, https://igordot.github.io/msigdbr/) to retrieve the pathway databases. The clusters were also scored for common cancer-related pathways using PROGENy (99).

Gene signatures for senescence and antigen presentation were compiled from various published signatures (100, 101) or manually curated (Supplementary Table S2). Specifically, senescent-like “Sen-High” tumor cells were defined as high for three signatures: a Cancer SENESCopedia signature (100), the SenMayo signature (101), and genes encoding SASP factors (Supplementary Table S2). Thresholding of Sen-High cells for each signature was determined by performing Gaussian mixture modeling. The thresholds were picked by modeling each senescence signature score as a mixture of two Gaussians, and the thresholds were set at the decision boundary of the two components of the Gaussian mixture model.

Plots were generated using R (version 4.4.1; June 14, 2024) and R visualization packages such as ComplexHeatmap (102) and scCustomize (https://doi.org/10.5281/zenodo.5706430).

### IHC and IF of FFPE Tissues

Tissues were fixed overnight in 10% formalin, embedded in paraffin, and cut into 5 µm sections. Hematoxylin and eosin staining was performed using standard protocols. For IHC and IF analysis of FFPE tissues, sections were deparaffinized, rehydrated, and boiled in a pressure cooker for 15 minutes in 10 mmol/L citrate buffer (pH 6.0) for antigen retrieval. Tissues were blocked and permeabilized in a block/perm solution (5% BSA and 0.3% Tween 20 in PBS). Primary antibodies were diluted in block/perm solution at the indicated concentrations and added to slides overnight at 4°C. The following primary antibodies were used: recombinant anti-CD4 antibody (EPR19514; Abcam, ab271945, 1:500). The next day, slides were washed in PBS and stained with secondary antibodies diluted in block/perm solution at the indicated concentrations for 4 to 6 hours. The following secondary antibodies were used: donkey anti-rabbit IgG (H + L) Highly Cross-Adsorbed Secondary Antibody, Alexa Fluor 555 (Thermo Fisher Scientific, cat. # A-31572, RRID:AB_162543, 1:500). Slides were washed and stained with directly conjugated antibodies diluted in block/perm solution at indicated concentrations overnight at 4°C. The following directly conjugated antibodies were used: CD8a (Abcam, cat. # ab277939, RRID:AB_3076317, clone EPR21769, Alexa Fluor 594, 1:500), Ki67 (Abcam, cat. # ab281928, clone SP6, Alexa Fluor 647, 1:500), pan-keratin (Cell Signaling Technology, cat. # 4523, RRID:AB_836889, clone C11, mouse mAb, Alexa Fluor 488, 1:100), and cleaved caspase 3 (Asp175; Cell Signaling Technology, cat. # 9664, RRID:AB_2070042, clone D3E9, rabbit mAb, Alexa Fluor 750, 1:100). The next day, slides were washed with PBS and stained with DAPI (1 μg/mL) diluted in PBS for 5 to 10 minutes. Finally, slides were washed, and coverslips were mounted using Fluoromount-G Mounting Medium (SouthernBiotech, 0100-01).

### IF Staining of 4% PFA-Fixed, OCT-Embedded Tissues

Pancreas tumor tissue was fixed in 4% PFA in PBS for 24 hours followed by cryoprotection in 30% sucrose for 4 hours and then embedded in OCT blocks on dry ice. OCT blocks were stored at −80°C. Ten-micrometer sections were cut using a cryostat. Slides were stored at −80°C. Prior to staining, slides were thawed for 15 minutes (or until moisture evaporated) at room temperature and then rehydrated in PBS. Tissue was outlined using an ImmEdge Hydrophobic Barrier PAP Pen. Tissue was blocked and permeabilized in a block/perm solution (5% BSA and 0.3% Tween 20 in PBS) for 20 minutes at room temperature. Primary antibodies were diluted in block/perm solution and added to slides overnight at 4°C. The following primary antibody was used: recombinant anti-phospho-p44/42 MAPK (Erk1/2) antibody (Thr202/Tyr204; Cell Signaling Technology, cat. # 4370, RRID:AB_2315112). The next day, slides were washed in PBS and stained with secondary antibodies diluted in block/perm solution at indicated concentrations for 4 to 6 hours. The following secondary antibodies were used: donkey anti-rabbit IgG (H + L) Highly Cross-Adsorbed Secondary Antibody, Alexa Fluor 594 (Thermo Fisher Scientific, cat. # A-21207, RRID:AB_141637, 1:500). Slides were washed and stained with directly conjugated antibodies diluted in block/perm solution at indicated concentrations overnight at 4°C. The following directly conjugated antibodies were used: CD45 (Novus Biologicals, cat. # NB100-77417AF750, RRID:AB_3172634, clone 30-F11, Alexa Fluor 750, 1:50), cleaved caspase-3 (Cell Signaling Technology, cat. # 9604, RRID:AB_2797708, clone D3E9, Alexa Fluor 555, 1:200), cleaved caspase-3 (Cell Signaling Technology, cat. # 97774, Alexa Fluor 750, 1:50), Ki67 (Abcam, cat. # ab281928, RRID:AB_3105924, clone SP6, Alexa Fluor 647, 1:500), CD3 (BD Biosciences, cat. # 557869, RRID:AB_39691, clone 17A2, Alexa Fluor 647, 1:200), CD4 (BioLegend, cat. # 100512, RRID:AB_312715, clone RM4-5, PE, 1:100), CD8a (Abcam, cat. # ab277939, RRID:AB_3076317, clone EPR21769, Alexa Fluor 594, 1:500), CD8a (Abcam, cat. # ab237365, clone EPR21769, Alexa Fluor 647, 1:500), CD11c (BioLegend, cat. # 117307, RRID:AB_313776, clone N418, PE, 1:100), B220 (BioLegend, cat. # 103226, RRID:AB_389330, clone RA3-6B2, Alexa Fluor 647, 1:100), MHC-II (I-A/I-E; BioLegend, cat. # 107650, RRID:AB_2566438, clone M5/114.15.2, 1:1000), podoplanin (BioLegend, cat. # 127407, RRID:AB_2161929, clone 8.1.1, PE, 1:5000), α-smooth muscle actin (Thermo Fisher Scientific, cat. # 50-9760-82, RRID:AB_2574362, clone 1A4, eFluor 660, 1:10,000), CD31 (BioLegend, cat. # 102520, RRID:AB_2563319, RRID:AB_2687065, clone MEC13.3, Alexa Fluor 594, 1:100), F4/80 (BioLegend, cat. # 123120, RRID:AB_893479, clone BM8, Alexa Fluor 488, 1:100), podoplanin (Novus Biologicals, cat. # NB600-1015AF750, RRID:AB_3192684, clone 8.1.1, AF750, 1:200), CD234 (e-cadherin; BioLegend, cat. # 147308, RRID:AB_2563955, Alexa Fluor 647, 1:100), CD234 (e-cadherin; Thermo Fisher Scientific, cat. # 50-112-4597 Alexa Fluor 488, 1:50), and myeloperoxidase (Abcam, cat. # ab225474, RRID:AB_2889205, Alexa Fluor 488, 1:500). The next day, slides were washed with PBS and stained with DAPI (1 μg/mL) diluted in PBS for 5 to 10 minutes. Finally, slides were washed, and coverslips were mounted using Fluoromount-G Mounting Medium (SouthernBiotech, 0100-01).

### SA-β-Gal Staining of 4% PFA-Fixed, OCT-Embedded Tissues

Prior to staining, slides were thawed for 15 minutes (or until moisture evaporated) at room temperature and then rehydrated in PBS. Tissue was outlined using an ImmEdge Hydrophobic Barrier PAP Pen. β-gal was detected using the chromogenic substrate X-gal (Invitrogen, 15520018) as follows. Tissues were washed with a room temperature 1 mmol/L MgCl_2_ PBS solution (pH 5.5, adjusted using HCl) 2 times. An X-gal working solution consisting of 1 mmol/L MgCl_2_ PBS (pH 5.5), 5 mmol/L potassium ferricyanide, 5 mmol/L potassium hexacyanoferrate (II), and X-gal (40× from 40 mg/mL stock diluted in N,N-dimethylformamide) was prepared and filtered through a Steriflip Vacuum Tube Top Filter (Millipore, SE1M179M6). The X-gal working solution was added to the slides and incubated at 37°C in a humidified chamber for 2 to 6 hours. Upon signal detection, the slides were washed in PBS 2×, and coverslips were mounted using Fluoromount-G Mounting Medium (SouthernBiotech, 0100-01).

### IF Imaging and Image Quantification

IF images were acquired as whole slide scans with a Pannoramic P250 Flash scanner (3DHISTECH) using a 20×/0.8 NA objective lens by the Molecular Cytology Core. For image quantification, whole tissue sections were used, or one to eight representative regions were randomly selected in 3DHISTECH SlideViewer (indicated in figure legends), avoiding areas of high background staining or out-of-focus tissue. Regions were exported as .tif files and quantified using ImageJ/Fiji (NIH) to measure the area and intensity of the fluorescent signal to classify cell positivity for each marker. For the quantification of lymphoid aggregates, the borders of B-cell follicles as indicated by dense regions of CD20 staining were outlined manually in 3DHISTECH SlideViewer (RRID:SCR_024885) to measure their area and the number of aggregates per tumor.

For highly multiplexed IF imaging of TLSs, RMC-7977 + palbociclib–treated tumors were subjected to sequential staining using the Leica Cell DIVE with dye inactivation between cycles (0.1 M NaHCO_3_, 3% H_2_O_2_). Screenshots were captured in QuPath software.

### Statistical Analyses

Statistical analyses were performed as described in the figure legend for each experiment. Group size was determined on the basis of the results of preliminary experiments, and no statistical method was used to predetermine sample size. The indicated sample size (*n*) represents biological replicates. All samples that met proper experimental conditions were included in the analysis. In particular, some tumor samples contained insufficient numbers of cells for flow cytometric analyses due to treatment-induced regressions. These samples were excluded from the analysis. Survival was measured using the Kaplan–Meier method. Statistical tests were performed using Prism 6 software (GraphPad Software) as indicated in the figure legends. Significance was set at *P* < 0.05. *P* values: ns, >0.05; *, ≤0.05; **, <0.01; ***, <0.001; ****, <0.0001.

### Data Availability

The data generated in this study are available within the article and its supplementary data files. scRNA-seq raw and processed data reported in this article are available upon request and were also deposited at the NCBI Gene Expression Omnibus in accordance with American Association for Cancer Research data availability policies under accession number GSE293456. All other raw data are available from the corresponding author upon reasonable request.

## Supplementary Material

Supplementary Table 1 Samples collected for single cell RNA-sequencing studies

Supplementary Table 2 Gene signatures used for single cell RNAseq analysis

Figure S1 Palbociclib increases the anti-tumor activity of RMC-7977

Figure S2 Palbociclib does not increase RASi-driven cell death or affect RASi-driven phospho ERK inhibition

Figure S3 Identification of tumor cell subsets expressing senescence gene signatures

Figure S4 Characterization of tumor immune infiltrates and immune dependency of anti-tumor activity following RASi + CDK4/6i treatment

Figure S5 CD40 agonist prolongs responses to RMC-7977 + palbociclib

Figure S6 Characterizing transcriptional changes in immune cells following RAS(ON) inhibitor-based combination strategies

Figure S7 Characterizing RAS(ON) inhibitor-based combination therapies in a differentiated mouse model of PDAC

Figure S8 Gating strategy for flow cytometry analysis in Fig 3A-B and Supplementary Fig S4A-N, S4Q-S.
