# Supplementary material for: A RAS(ON) Multi-Selective Inhibitor Combination Therapy Triggers Long-term Tumor Control through Senescence-Associated Tumor-Immune Equilibrium in Pancreatic Ductal Adenocarcinoma
Source: Cancer Discov. 2025 Apr 29;15(8):1717–39. doi: 10.1158/2159-8290.CD-24-1425 (PMC12319406; doi:10.1158/2159-8290.CD-24-1425)
Supplement: Figure S1 — Palbociclib increases the anti-tumor activity of RMC-7977 [file cd-24-1425_figure_s1_suppsf1.pdf]

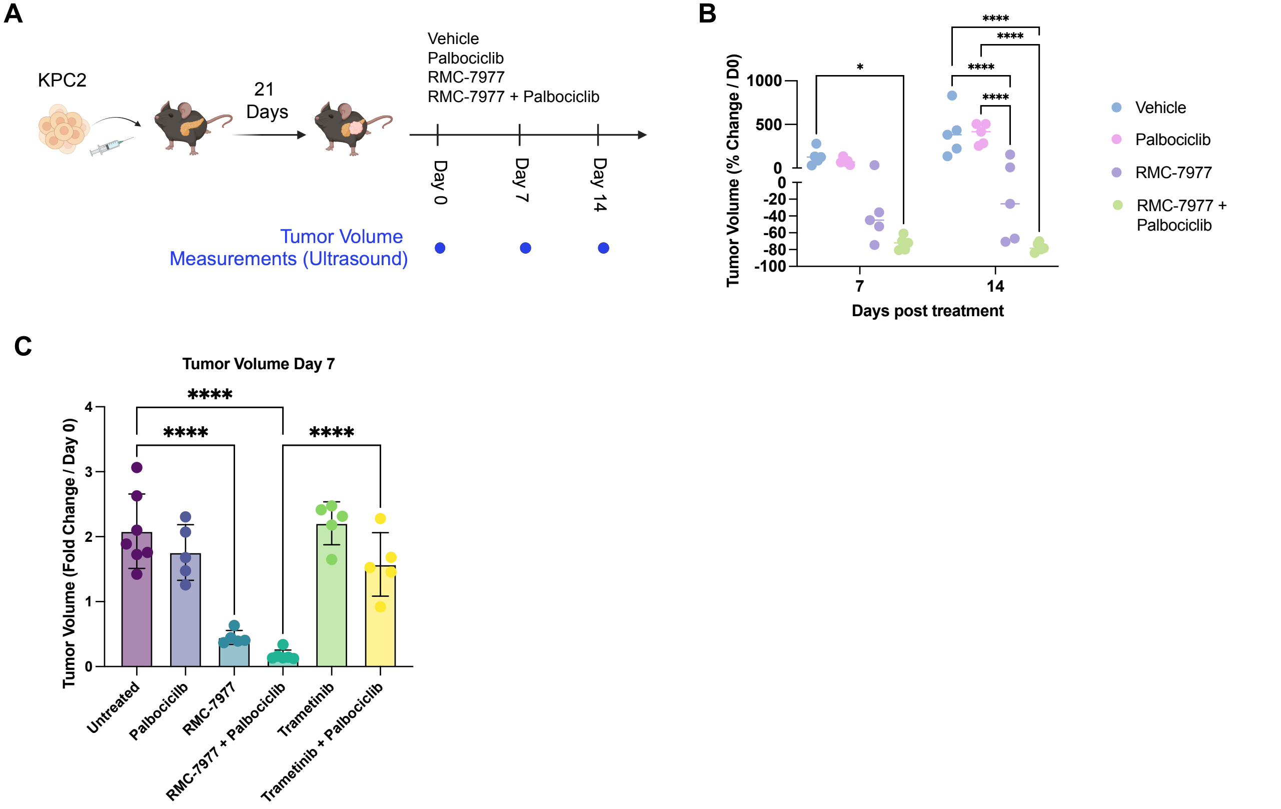

Supplementary Figure S1: Palbociclib increases the anti-tumor activity of RMC-7977

(A) Scheme of experimental design (KPC2 orthotopic transplant into wildtype C57Bl/6 mice) (Created with BioRender.com).

(B) Percent change in tumor volume (y axis) at indicated time points (x axis) compared to day 0 as measured by weekly ultrasound. Each dot represents an individual mouse. Statistical testing: a Two-way ANOVA was performed comparing the mean of each treatment group with every other treatment group within each time point, correcting for multiple comparisons with a Tukey test. All statistically significant comparisons are shown.

(C) Percent change in tumor volume (y axis) compared to day 0 as measured by weekly ultrasound. Each dot represents an individual mouse. Statistical testing: Ordinary one-way ANOVA, comparing the mean of each treatment group with the mean of every other treatment group, correcting for multiple comparisons with Tukey test. Only relevant statistically significant comparisons are shown.
