# Supplementary material for: A RAS(ON) Multi-Selective Inhibitor Combination Therapy Triggers Long-term Tumor Control through Senescence-Associated Tumor-Immune Equilibrium in Pancreatic Ductal Adenocarcinoma
Source: Cancer Discov. 2025 Apr 29;15(8):1717–39. doi: 10.1158/2159-8290.CD-24-1425 (PMC12319406; doi:10.1158/2159-8290.CD-24-1425)
Supplement: Figure S2 — Palbociclib does not increase RASi-driven cell death or affect RASi-driven phospho ERK inhibition [file cd-24-1425_figure_s2_suppsf2.pdf]

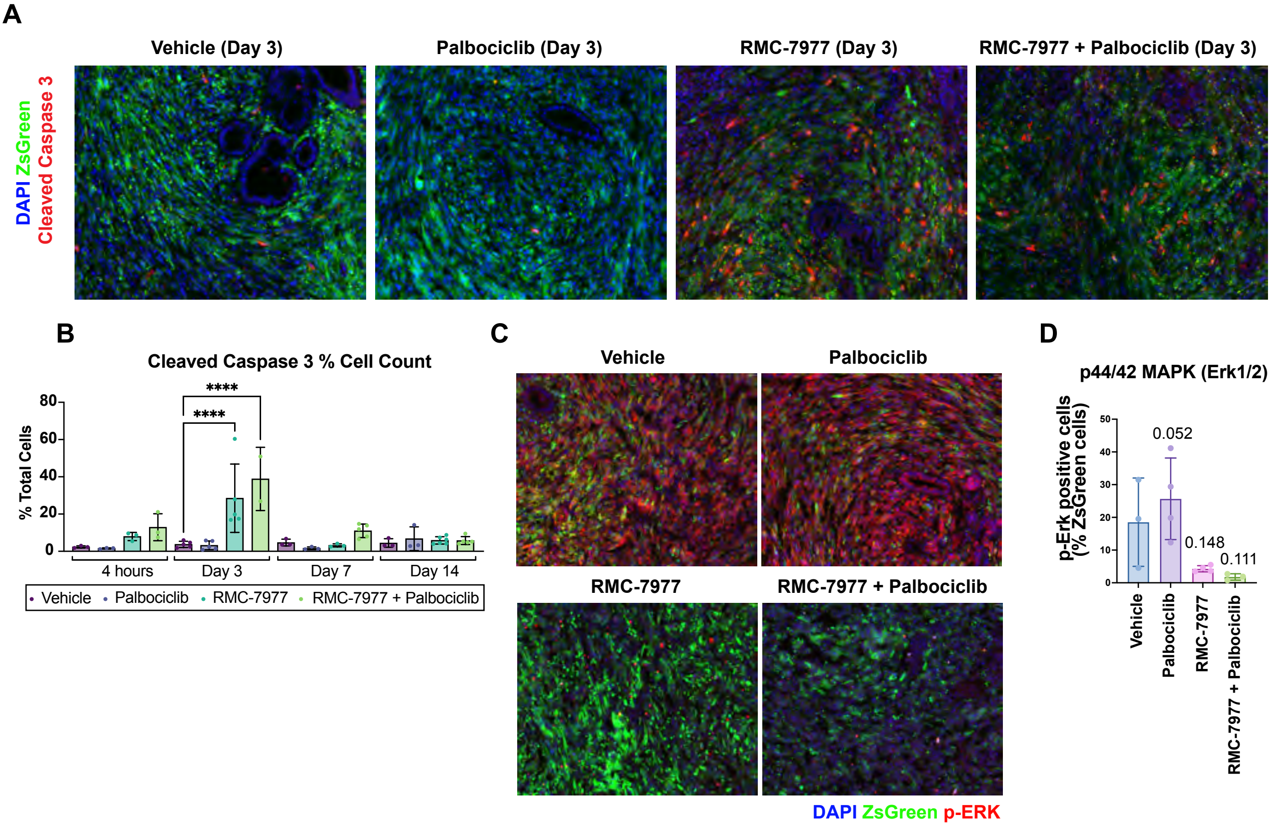

Supplementary Figure S2: Palbociclib does not increase RASi-driven cell death or affect RASi-driven phospho ERK inhibition

(A) Representative images showing apoptotic cells as indicated by cleaved caspase-3 staining (red). zsGreen (green) marks tumor cells and DAPI (blue) marks nuclei.

(B) Quantification of immunofluorescence staining of pancreatic tumor tissues for Cleaved Caspase 3 in mice 4 hours, 3 days, 7 days, or 14 days post treatment initiation. Each dot represents an individual mouse (average of 3-5 ~40,000 µm2 regions). Statistical testing: Ordinary one-way ANOVA, comparing preselected pairs of columns (within treatment timepoint vs. vehicle only), correcting for multiple comparisons with a Bonferroni test. Only statistically significant comparisons are shown.

(C) Representative images showing Phospho-p44/42 MAPK (Erk1/2) (p-ERK) staining (red) in tumors collected 4 hours after first dose. ZsGreen (green) marks tumor cells and DAPI (blue) marks nuclei.

(D) Quantification of immunofluorescence staining of pancreatic tumor tissues for p-ERK in mice 4 hours post treatment initiation. Each dot represents an individual mouse (whole tissue quantified). Statistical testing: Ordinary one-way ANOVA, comparing the mean of every column with the control column (Vehicle), correcting for multiple comparisons with a Dunnet test. Relevant p values are shown.
