# Supplementary material for: A RAS(ON) Multi-Selective Inhibitor Combination Therapy Triggers Long-term Tumor Control through Senescence-Associated Tumor-Immune Equilibrium in Pancreatic Ductal Adenocarcinoma
Source: Cancer Discov. 2025 Apr 29;15(8):1717–39. doi: 10.1158/2159-8290.CD-24-1425 (PMC12319406; doi:10.1158/2159-8290.CD-24-1425)
Supplement: Figure S3 — Identification of tumor cell subsets expressing senescence gene signatures [file cd-24-1425_figure_s3_suppsf3.pdf]

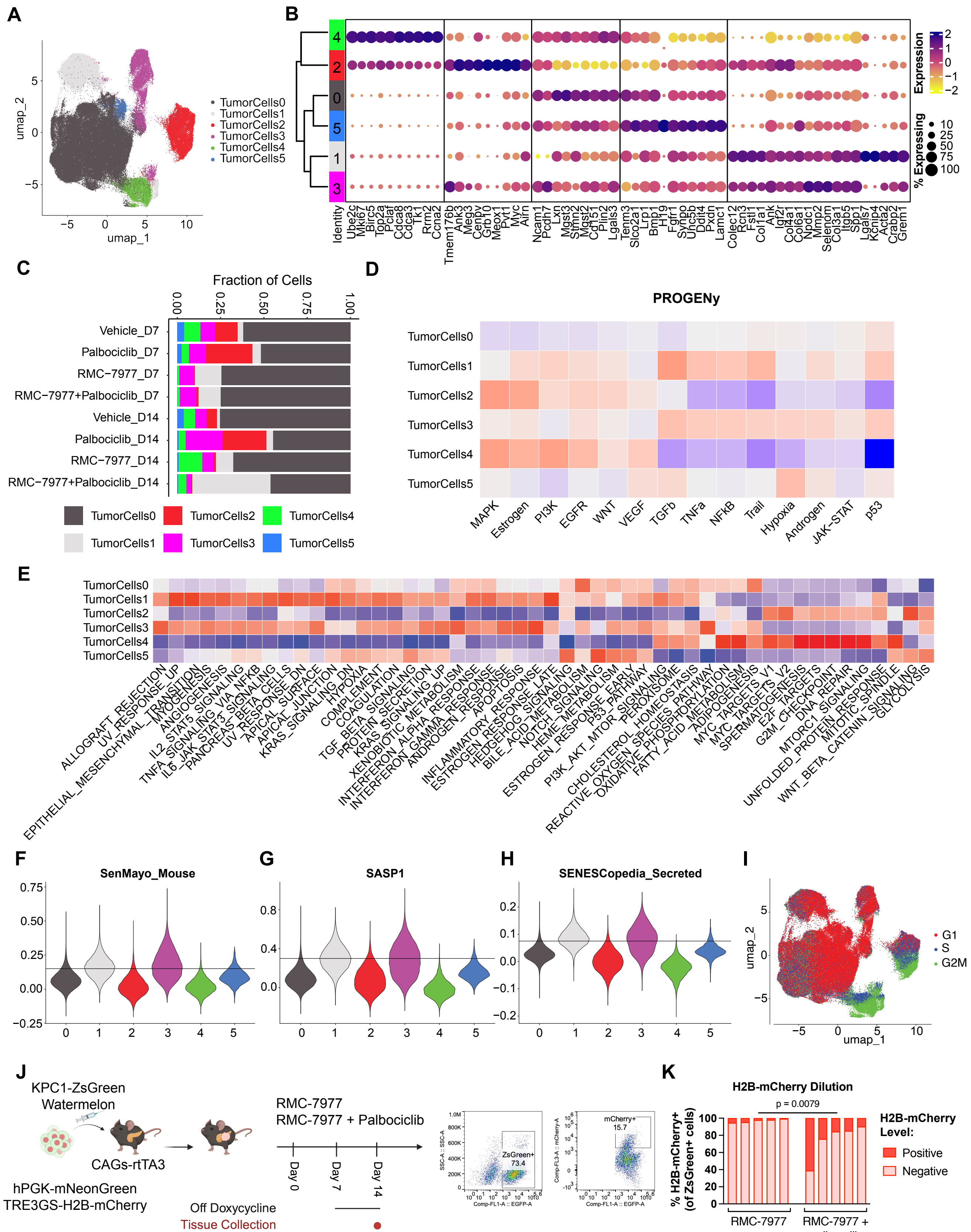
A
B
C
D
E
F
G
H
I
J
K
umap_1
umap_2
TumorCells0
TumorCells1
TumorCells2
TumorCells3
TumorCells4
TumorCells5
Identity
Expression
% Expressing
Fraction of Cells
Vehicle_D7
Palbociclib_D7
RMC−7977_D7
RMC−7977+Palbociclib_D7
Vehicle_D14
Palbociclib_D14
RMC−7977_D14
RMC−7977+Palbociclib_D14
PROGENy
MAPK
Estrogen
PI3K
EGFR
WNT
VEGF
TGFb
TNFa
NFkB
Trail
Hypoxia
Androgen
JAK-STAT
p53
SenMayo_Mouse
SASP1
SENESCopedia_Secreted
G1
S
G2M
KPC1-ZsGreen
Watermelon
CAGs-rtTA3
hPGK-mNeonGreen
TRE3GS-H2B-mCherry
RMC-7977
RMC-7977 + Palbociclib
Day 0
Day 7
Day 14
Off Doxycycline
Tissue Collection
ZsGreen+
73.4
mCherry+
15.7
H2B-mCherry Dilution
p = 0.0079
% H2B-mCherry+ (of ZsGreen+ cells)
H2B-mCherry Level:
Positive
Negative
RMC-7977
RMC-7977 + palbcoclib

Sup Fig 3

Supplementary Figure S3: Identification of tumor cell subsets expressing senescence gene signatures

(A) UMAP showing Seurat clustering of single cell RNA-sequencing of ZsGreen+ sorted KPC1 tumor cells.

(B) Dot plot showing cluster-defining marker genes.

(C) Fraction of tumor cells from each treatment group residing in each cluster.

(D) PROGENy analysis of clusters.

(E) Heatmap showing Hallmark signature scores in each cluster.

(F) Violin plot of SenMayo_Mouse gene expression across Seurat clusters. Threshold shows high vs. low expression as determined by a Gaussian Mixture Model.

(G) Violin plot of SASP gene expression across Seurat clusters. Threshold shows high vs. low expression as determined by a Gaussian Mixture Model.

(H) Violin plot of SENEScopedia_Secreted gene expression across Seurat clusters. Threshold shows high vs. low expression as determined by a Gaussian Mixture Model.

(I) UMAP visualization of cells colored by cell cycle phase (G1, S and G2/M). Cells were assigned to phases based on Cell Cycle Scoring.

(J) Scheme of experimental design (KPC1-ZsGreen-Watermelon orthotopic transplant into Rosa26-CAGs-rtTA3 mice) to trace proliferative history of tumor cells following indicated treatments between 7 and 14 days of treatment. Strategy for gating DAPI-, CD45-, ZsGreen+ tumor cells for H2B-mCherry positivity is shown.

(K) Fraction of tumor cells with positive (arrested since 7 days post treatment initiation), or no H2B-mCherry following indicated treatments. Each bar represents an individual mouse. Statistical testing: Unpaired, nonparametric Mann-Whitney test, comparing the fraction of H2B-mCherry positive cells between RMC-7977 (n=5) and RMC-7977 + palbociclib (n=5) treated mice. Two-tailed P value is shown.
