# Supplementary material for: A RAS(ON) Multi-Selective Inhibitor Combination Therapy Triggers Long-term Tumor Control through Senescence-Associated Tumor-Immune Equilibrium in Pancreatic Ductal Adenocarcinoma
Source: Cancer Discov. 2025 Apr 29;15(8):1717–39. doi: 10.1158/2159-8290.CD-24-1425 (PMC12319406; doi:10.1158/2159-8290.CD-24-1425)
Supplement: Figure S4 — Characterization of tumor immune infiltrates and immune dependency of anti-tumor activity following RASi + CDK4/6i treatment [file cd-24-1425_figure_s4_suppsf4.pdf]

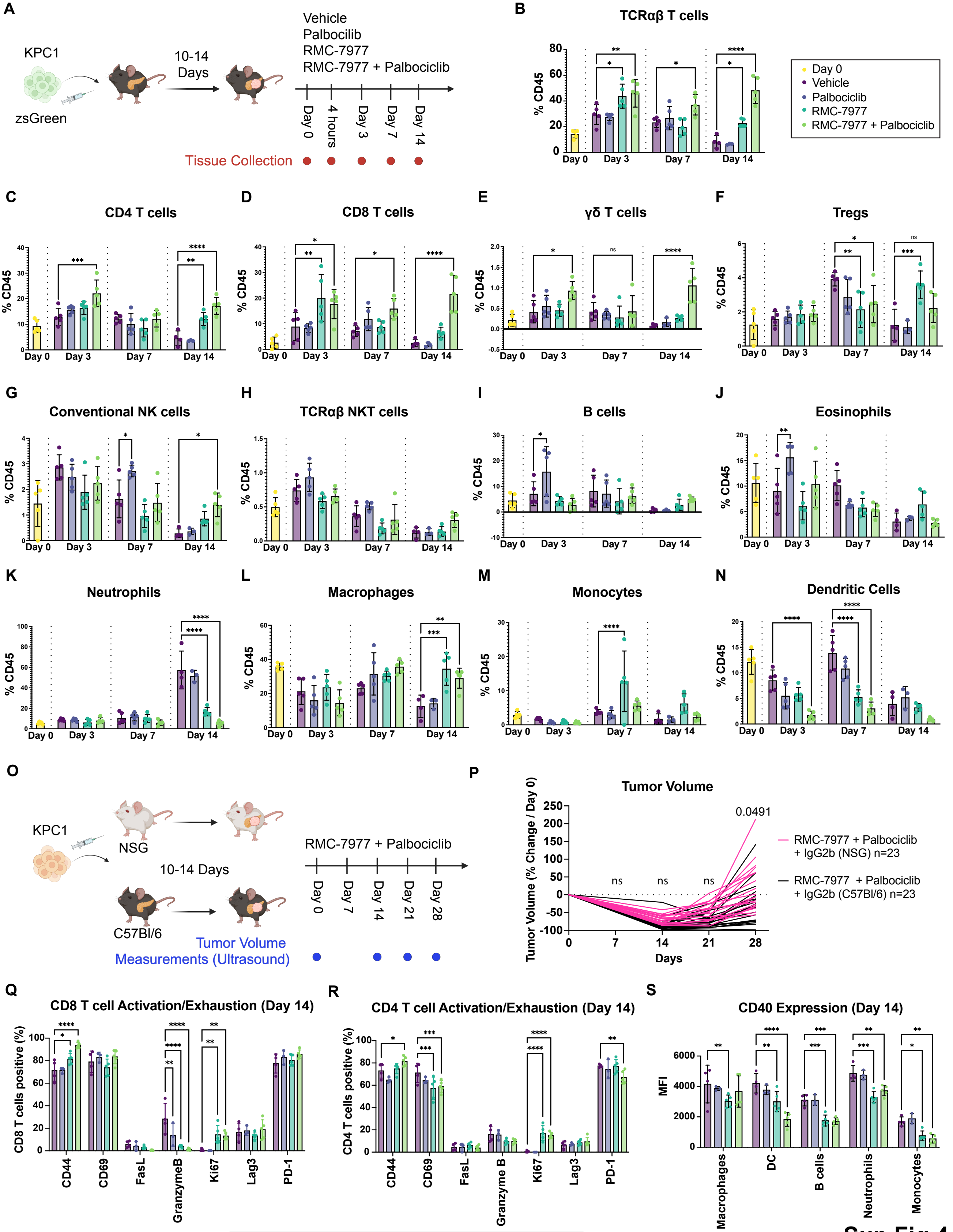

A
KPC1
zsGreen
10-14 Days
Vehicle
Palbocilib
RMC-7977
RMC-7977 + Palbociclib
Day 0
4 hours
Day 3
Day 7
Day 14
Tissue Collection
B
TCRαβ T cells
% CD45
Day 0
Vehicle
Palbociclib
RMC-7977
RMC-7977 + Palbociclib
C
CD4 T cells
D
CD8 T cells
E
γδ T cells
F
Tregs
G
Conventional NK cells
H
TCRαβ NKT cells
I
B cells
J
Eosinophils
K
Neutrophils
L
Macrophages
M
Monocytes
N
Dendritic Cells
O
KPC1
NSG
10-14 Days
C57Bl/6
RMC-7977 + Palbociclib
Day 0
Day 7
Day 14
Day 21
Day 28
Tumor Volume
Measurements (Ultrasound)
P
Tumor Volume
Tumor Volume (% Change / Day 0)
Days
0.0491
ns
RMC-7977 + Palbociclib + IgG2b (NSG) n=23
RMC-7977 + Palbociclib + IgG2b (C57Bl/6) n=23
Q
CD8 T cell Activation/Exhaustion (Day 14)
CD8 T cells positive (%)
CD44
CD69
FasL
GranzymeB
Ki67
Lag3
PD-1
R
CD4 T cell Activation/Exhaustion (Day 14)
CD4 T cells positive (%)
Granzyme B
S
CD40 Expression (Day 14)
MFI
Macrophages
DC
B cells
Neutrophils
Monocytes
Vehicle
Palbociclib
RMC-7977
RMC-7977 + Palbociclib

Sup Fig 4

Supplementary Figure S4. Characterization of tumor immune infiltrates and immune dependency of anti-tumor activity following RASi + CDK4/6i treatment

(A) Scheme of experimental design (KPC1-ZsGreen orthotopic transplant into wildtype C57Bl/6 mice) (Created with BioRender.com).
(B) Fraction of TCRβ+ T cells out of total CD45 cells by flow cytometry at indicated time-points following indicated treatments. Each dot represents an individual mouse. Legend applies to Figure S4B-N.
(C) Fraction of CD4+ T cells out of total CD45 cells by flow cytometry at indicated time-points following indicated treatments. Each dot represents an individual mouse.
(D) Fraction of CD8+ T cells out of total CD45 cells by flow cytometry at indicated time-points following indicated treatments. Each dot represents an individual mouse.
(E) Fraction of γδ T cells out of total CD45 cells by flow cytometry at indicated timepoints following indicated treatments. Each dot represents an individual mouse.
(F) Fraction of Foxp3+,CD25+ Tregs out of total CD45 cells by flow cytometry at indicated timepoints following indicated treatments. Each dot represents an individual mouse.
(G) Fraction of NK1.1+ conventional NK cells out of total CD45 cells by flow cytometry at indicated timepoints following indicated treatments. Each dot represents an individual mouse.
(H) Fraction of TCRβ+ NKT cells out of total CD45 cells by flow cytometry at indicated timepoints following indicated treatments. Each dot represents an individual mouse.
(I) Fraction of CD20+, MHC-II+ B cells out of total CD45 cells by flow cytometry at indicat-ed timepoints following indicated treatments. Each dot represents an individual mouse.
(J) Fraction of Siglec F+ eosinophils out of total CD45 cells by flow cytometry at indicated timepoints following indicated treatments. Each dot represents an individual mouse.
(K) Fraction of CD11b+, Ly-6g+ neutrophils out of total CD45 cells by flow cytometry at indicated timepoints following indicated treatments. Each dot represents an individual mouse.
(L) Fraction of F4/80+ macrophages out of total CD45 cells by flow cytometry at indicated timepoints following indicated treatments. Each dot represents an individual mouse.
(M) Fraction of CD11b+, Ly-6C+ monocytes out of total CD45 cells by flow cytometry at indicated timepoints following indicated treatments. Each dot represents an individual mouse.
(N) Fraction of CD11c+, MHC-II+ dendritic cells out of total CD45 cells by flow cytometry at indicated timepoints following indicated treatments. Each dot represents an individual mouse.

Statistical testing for B-N: Ordinary one-way ANOVA with multiple comparisons comparing the means of preselected pairs of columns (each treatment group against the vehicle of the relevant time point), and correcting for multiple comparisons using a Sidak test. Only statistically significant comparisons are shown.

Supplementary Figure S4. Characterization of tumor immune infiltrates and immune dependency of anti-tumor activity following RASi + CDK4/6i treatment (continued)

(O) Scheme of experimental design (KPC1 orthotopic transplant into wildtype C57Bl/6 or NSG mice) (Created with BioRender.com).
(P) Percent change (over baseline) in tumor volume of C57Bl/6 mice (n=23 at day 0, n=20 at day 28) or NSG mice (n=23 at day 0, n=19 at day 28) treated with RMC-7977 + Palbociclib + IgG2b. Each line represents an individual mouse. Statistical testing: Two-way ANOVA comparing mouse strains within each timepoint), with Bonferroni testing to correct for multiple comparisons. Statistically significant p values are shown.
(Q) Fraction of CD8 T cells (%) positive for indicated markers at 14 days post treatment initiation. Each dot represents an individual mouse.
(R) Fraction of CD4 T cells (%) positive for indicated markers at 14 days post treatment initiation. Each dot represents an individual mouse.
(S) Mean Fluorescence Intensity (MFI) of surface CD40 levels on indicated immune cell subsets at 14 days post treatment initiation. Each dot represents an individual mouse.

Statistical testing for Q-S: Two-way ANOVA comparing each treatment group to vehicle within each marker (Q-R) or cell type (S), with Dunnett testing to correct for multiple comparisons. Statistically significant p values are shown.
