# Supplementary material for: A RAS(ON) Multi-Selective Inhibitor Combination Therapy Triggers Long-term Tumor Control through Senescence-Associated Tumor-Immune Equilibrium in Pancreatic Ductal Adenocarcinoma
Source: Cancer Discov. 2025 Apr 29;15(8):1717–39. doi: 10.1158/2159-8290.CD-24-1425 (PMC12319406; doi:10.1158/2159-8290.CD-24-1425)
Supplement: Figure S5 — CD40 agonist prolongs responses to RMC-7977 + palbociclib [file cd-24-1425_figure_s5_suppsf5.pdf]

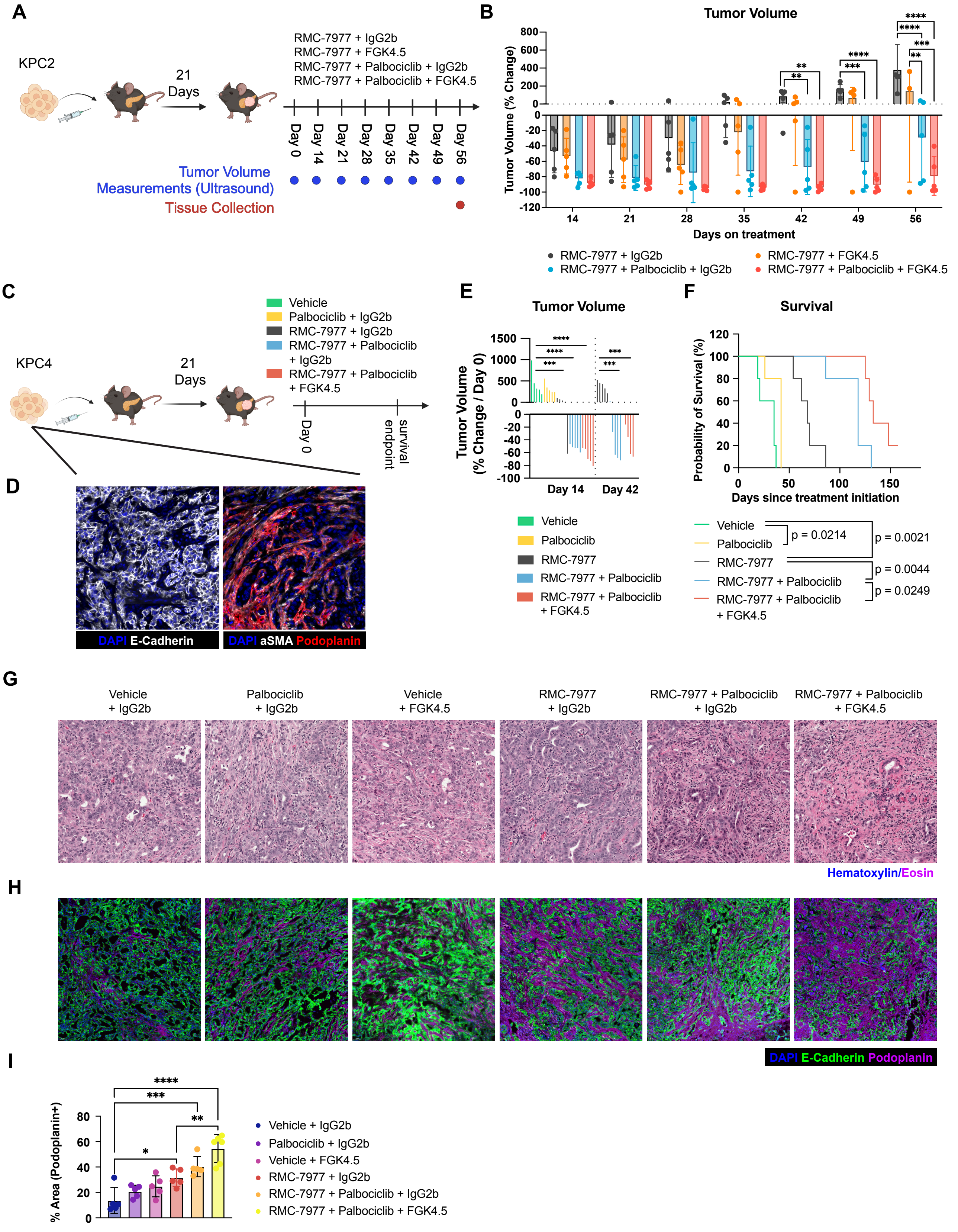
A
KPC2
21 Days
RMC-7977 + IgG2b
RMC-7977 + FGK4.5
RMC-7977 + Palbociclib + IgG2b
RMC-7977 + Palbociclib + FGK4.5
Day 0
Day 14
Day 21
Day 28
Day 35
Day 42
Day 49
Day 56
Tumor Volume Measurements (Ultrasound)
Tissue Collection
B
Tumor Volume
Tumor Volume (% Change)
Days on treatment
RMC-7977 + IgG2b
RMC-7977 + FGK4.5
RMC-7977 + Palbociclib + IgG2b
RMC-7977 + Palbociclib + FGK4.5
C
KPC4
21 Days
Vehicle
Palbociclib + IgG2b
RMC-7977 + IgG2b
RMC-7977 + Palbociclib + IgG2b
RMC-7977 + Palbociclib + FGK4.5
Day 0
survival endpoint
D
DAPI E-Cadherin
DAPI aSMA Podoplanin
E
Tumor Volume
Tumor Volume (% Change / Day 0)
Day 14
Day 42
Vehicle
Palbociclib
RMC-7977
RMC-7977 + Palbociclib
RMC-7977 + Palbociclib + FGK4.5
F
Survival
Probability of Survival (%)
Days since treatment initiation
Vehicle
Palbociclib
RMC-7977
RMC-7977 + Palbociclib
RMC-7977 + Palbociclib + FGK4.5
p = 0.0214
p = 0.0021
p = 0.0044
p = 0.0249
G
Vehicle + IgG2b
Palbociclib + IgG2b
Vehicle + FGK4.5
RMC-7977 + IgG2b
RMC-7977 + Palbociclib + IgG2b
RMC-7977 + Palbociclib + FGK4.5
Hematoxylin/Eosin
H
DAPI E-Cadherin Podoplanin
I
% Area (Podoplanin+)
Vehicle + IgG2b
Palbociclib + IgG2b
Vehicle + FGK4.5
RMC-7977 + IgG2b
RMC-7977 + Palbociclib + IgG2b
RMC-7977 + Palbociclib + FGK4.5

Sup Fig 5

Supplementary Figure S5. CD40 agonist prolongs responses to RMC-7977 + Palbociclib

(A) Scheme of experimental design (KPC2 orthotopic transplant into wildtype C57Bl/6 mice) (Created with BioRender.com).

(B) Percent change in tumor volume compared to day 0 as measured by weekly ultrasound. The y axis indicates the % change in tumor volume and the x axis indicates the time point post treatment initiation. Each dot represents an individual mouse (n=5 per treatment group at treatment initiation) Statistical testing: Two-way ANOVA with multiple comparisons (comparing each treatment group against every other treatment group within the same timepoint), with Tukey testing to correct for multiple comparisons. Only statistically significant comparisons are shown. At 49 days, n=4 RMC-7977 + FGK4.5 mice remained, and n=5 remained for all other treatment groups. At 56 days, n= 3 RMC-7977 + FGK4.5 mice remained, and n=5 remained for all other treatment groups.

(C) Scheme of experimental design (KPC4 orthotopic transplant into wildtype C57Bl/6 mice) (Created with BioRender.com).

(D) Representative image showing staining for the epithelial marker E-cadherin (marks tumor cells), the fibroblast marker Podoplanin and the activated fibroblast marker alpha Smooth Muscle Actin in KPC4 derived tumors following 21 days of engraftment.

(E) Percent change in tumor volume compared to baseline at 14 or 42 days post treatment initiation following treatment with indicated agents. Statistical testing: Ordinary one-way ANOVA, comparing the mean of each treatment group with the mean of every other treatment group in the same timepoint, correcting for multiple comparisons with the Bonferroni test. Only relevant statistically significant comparisons are shown. Sample size: n=5 for each group.

(F) Probability of survival (%) following treatment with indicated agents. Statistical testing: A log-rank (Mantel-Cox) test was performed to compare survival curves and relevant p values are shown. Sample size: n=5 for each group.

(G) Representative snapshots of H&E stained KPC4-derived tumors following 7 days of treatment with indicated agents. Tumor tissue was harvested 2 hours after mice received their final dose.

(H) Representative snapshots of podoplanin and E-cadherin stained KPC4-derived tumors following 7 days of treatment with indicated agents.

(I) Quantification of representative regions of immunofluorescence staining for podoplanin+ area as a percentage of total area (representative images shown in Sup Fig 5H) in tumors 7 days post treatment initiation (average of 3-5 ~30,000 μm2 regions). Each dot represents an individual mouse (n=5 for each treatment group, with the exception of RMC-7977 + palbociclib + FGK4.5 for which n=6). Statistical testing: One-way ANOVA comparing the mean of every column with the mean of every other column, correcting for multiple comparisons with a Tukey test. All relevant statistically significant comparisons are shown.
