# Supplementary material for: A RAS(ON) Multi-Selective Inhibitor Combination Therapy Triggers Long-term Tumor Control through Senescence-Associated Tumor-Immune Equilibrium in Pancreatic Ductal Adenocarcinoma
Source: Cancer Discov. 2025 Apr 29;15(8):1717–39. doi: 10.1158/2159-8290.CD-24-1425 (PMC12319406; doi:10.1158/2159-8290.CD-24-1425)
Supplement: Figure S6 — Characterizing transcriptional changes in immune cells following RAS(ON) inhibitor-based combination strategies [file cd-24-1425_figure_s6_suppsf6.pdf]

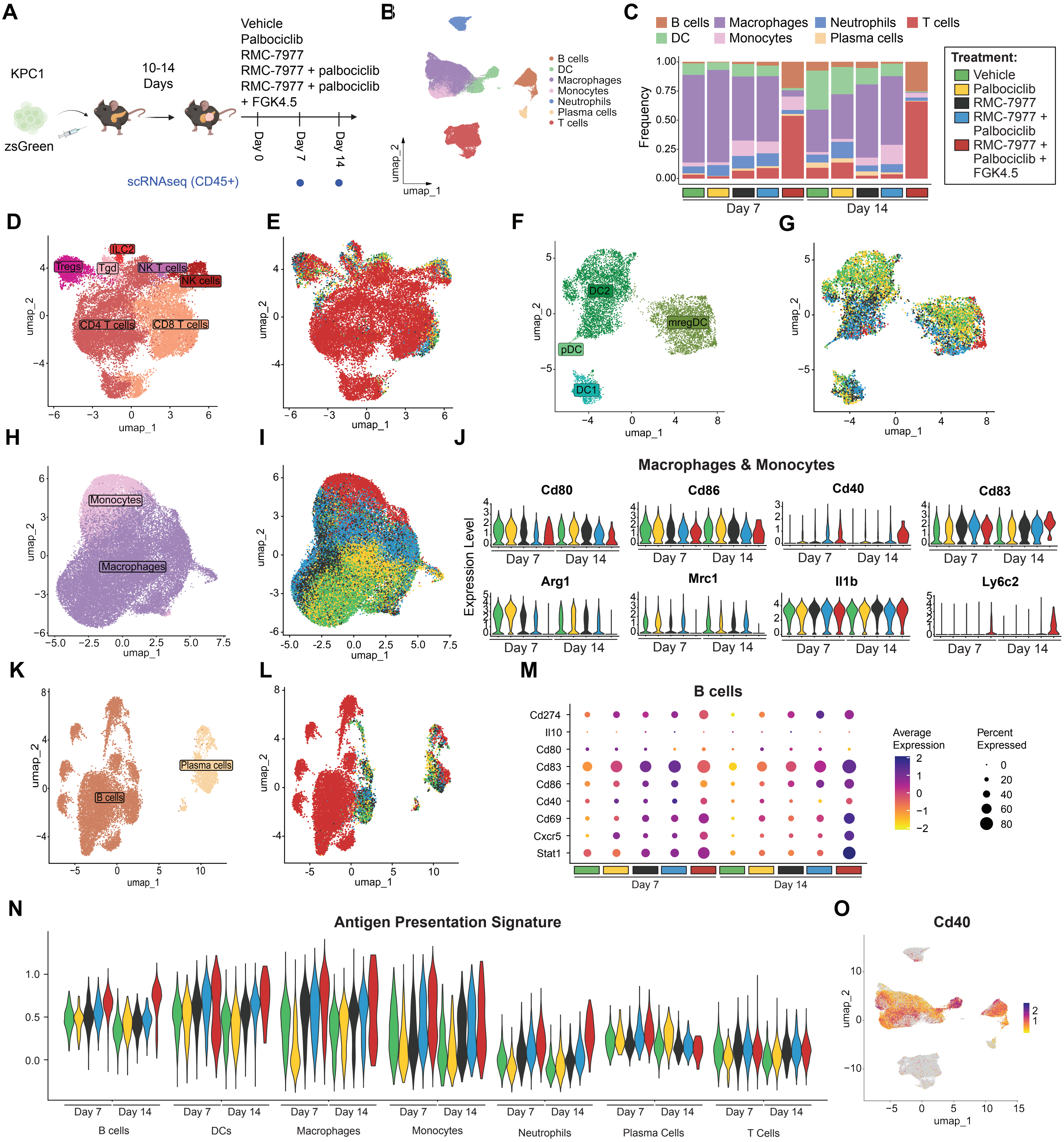
A
KPC1
zsGreen
10-14 Days
Vehicle
Palbociclib
RMC-7977
RMC-7977 + palbociclib
RMC-7977 + palbociclib + FGK4.5
Day 0
Day 7
Day 14
scRNAseq (CD45+)
B
B cells
DC
Macrophages
Monocytes
Neutrophils
Plasma cells
T cells
umap_1
umap_2
C
Frequency
Treatment:
Vehicle
Palbociclib
RMC-7977
RMC-7977 + Palbociclib
RMC-7977 + Palbociclib + FGK4.5
Day 7
Day 14
D
Tregs
ILC2
Tgd
NK T cells
NK cells
CD4 T cells
CD8 T cells
E
F
DC2
mregDC
pDC
DC1
G
H
Monocytes
Macrophages
I
J
Macrophages & Monocytes
Expression Level
Cd80
Cd86
Cd40
Cd83
Arg1
Mrc1
Il1b
Ly6c2
K
Plasma cells
B cells
L
M
B cells
Cd274
Il10
Cd80
Cd83
Cd86
Cd40
Cd69
Cxcr5
Stat1
Average Expression
Percent Expressed
N
Antigen Presentation Signature
B cells
DCs
Macrophages
Monocytes
Neutrophils
Plasma Cells
T Cells
O
Cd40

Sup Fig 6

Supplementary Figure S6. Characterizing transcriptional changes in immune cells following RAS(ON) inhibitor-based combination strategies

(A) Scheme of experimental design for single cell RNA-sequencing of CD45+ immune cells (KPC1 orthotopic transplant into wildtype C57Bl/6 mice) (Created with BioRender.com).
(B) UMAP of CD45+ immune cells.
(C) Distribution of immune cell populations following indicated treatments. Legend indicating color scheme for treatment groups applies to Sup Fig 6E-G, I-J, L, N.
(D) UMAP of T cells, NK cells and Group 2 innate lymphoid cells (ILC2s) colored by cell subtype.
(E) UMAP of T cells, NK cells and Group 2 innate lymphoid cells (ILC2s) colored by treatment group.
(F) UMAP of DCs colored by cell subtype.
(G) UMAP of DCs colored by treatment group.
(H) UMAP of macrophages and monocytes colored by cell subtype.
(I) UMAP of macrophages and monocytes colored by treatment group.
(J) Violin plots of macrophage and monocyte expression of indicated genes.
(K) UMAP of B cells and plasma cells colored by cell subtype.
(L) UMAP of B cells and plasma cells colored by treatment group.
(M) Dot plot of B cell expression of indicated genes.
(N) Violin plot of antigen presentation gene signature in indicated immune cell subtypes.
(O) UMAP of Cd40 expression across all CD45+ immune cells.
