# Supplementary material for: A RAS(ON) Multi-Selective Inhibitor Combination Therapy Triggers Long-term Tumor Control through Senescence-Associated Tumor-Immune Equilibrium in Pancreatic Ductal Adenocarcinoma
Source: Cancer Discov. 2025 Apr 29;15(8):1717–39. doi: 10.1158/2159-8290.CD-24-1425 (PMC12319406; doi:10.1158/2159-8290.CD-24-1425)
Supplement: Figure S7 — Characterizing RAS(ON) inhibitor-based combination therapies in a differentiated mouse model of PDAC [file cd-24-1425_figure_s7_suppsf7.pdf]

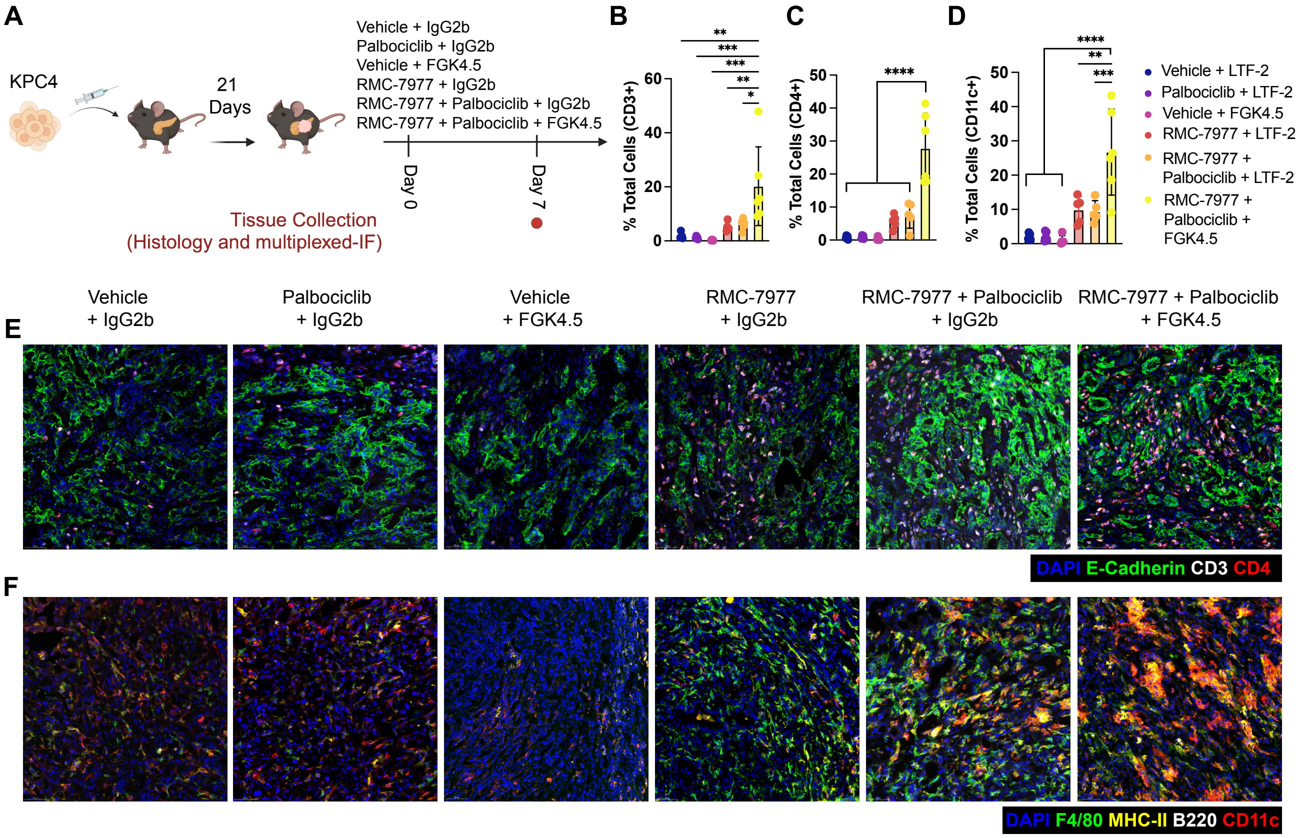
A
KPC4
21 Days
Vehicle + IgG2b
Palbociclib + IgG2b
Vehicle + FGK4.5
RMC-7977 + IgG2b
RMC-7977 + Palbociclib + IgG2b
RMC-7977 + Palbociclib + FGK4.5
Day 0
Day 7
Tissue Collection
(Histology and multiplexed-IF)
B
% Total Cells (CD3+)
C
% Total Cells (CD4+)
D
% Total Cells (CD11c+)
Vehicle + LTF-2
Palbociclib + LTF-2
Vehicle + FGK4.5
RMC-7977 + LTF-2
RMC-7977 + Palbociclib + LTF-2
RMC-7977 + Palbociclib + FGK4.5
E
Vehicle + IgG2b
Palbociclib + IgG2b
Vehicle + FGK4.5
RMC-7977 + IgG2b
RMC-7977 + Palbociclib + IgG2b
RMC-7977 + Palbociclib + FGK4.5
DAPI E-Cadherin CD3 CD4
F
DAPI F4/80 MHC-II B220 CD11c

Supp Fig 7

Supplementary Figure S7. Characterizing RAS(ON) inhibitor-based combination therapies in a differentiated mouse model of PDAC
(A) Scheme of experimental design (KPC4 orthotopic transplant into wildtype C57Bl/6 mice). Tumor tissue was harvested 7 days after mice received their first dose and 2 hours after mice received their last dose (Created with BioRender.com).
(B) Quantification of representative regions of immunofluorescence staining for CD3+ cell counts as a percentage of total cells (representative images shown in Sup Fig 7E) in tumors (average of 3-5 ~30,000 μm2 regions). Each dot represents an individual mouse (n=5 for each treatment group, with the exception of RMC-7977 + palbociclib + FGK4.5 for which n=6).
(C) Quantification of representative regions of immunofluorescence staining for CD4+ cell counts as a percentage of total cells (representative images shown in Sup Fig 7E) in tumors (average of 3-5 ~30,000 μm2 regions). Each dot represents an individual mouse (n=5 for each treatment group, with the exception of RMC-7977 + palbociclib + FGK4.5 for which n=6).
(D) Quantification of representative regions of immunofluorescence staining for CD11c+ cell counts as a percentage of total cells (representative images shown in Sup Fig 7F) in tumors (average of 3-5 ~30,000 μm2 regions). Each dot represents an individual mouse (n=5 for each treatment group, with the exception of RMC-7977 + palbociclib + FGK4.5 for which n=6).

Statistical testing for B-D: One-way ANOVA comparing the mean of every treatment group with the mean of every other treatment group, correcting for multiple comparisons with a Tukey test. All comparisons are shown.

(E) Representative snapshots of E-cadherin, CD3 and CD4 stained KPC4-derived tumors following 7 days of treatment with indicated agents.
(F) Representative snapshots of F4/80, MHC-II, B220 and CD11c stained KPC4-derived tumors following 7 days of treatment with indicated agents.
