# Supplementary material for: A RAS(ON) Multi-Selective Inhibitor Combination Therapy Triggers Long-term Tumor Control through Senescence-Associated Tumor-Immune Equilibrium in Pancreatic Ductal Adenocarcinoma
Source: Cancer Discov. 2025 Apr 29;15(8):1717–39. doi: 10.1158/2159-8290.CD-24-1425 (PMC12319406; doi:10.1158/2159-8290.CD-24-1425)
Supplement: Figure S8 — Gating strategy for flow cytometry analysis in Fig 3A-B and Supplementary Fig S4A-N, S4Q-S. [file cd-24-1425_figure_s8_suppsf8.pdf]

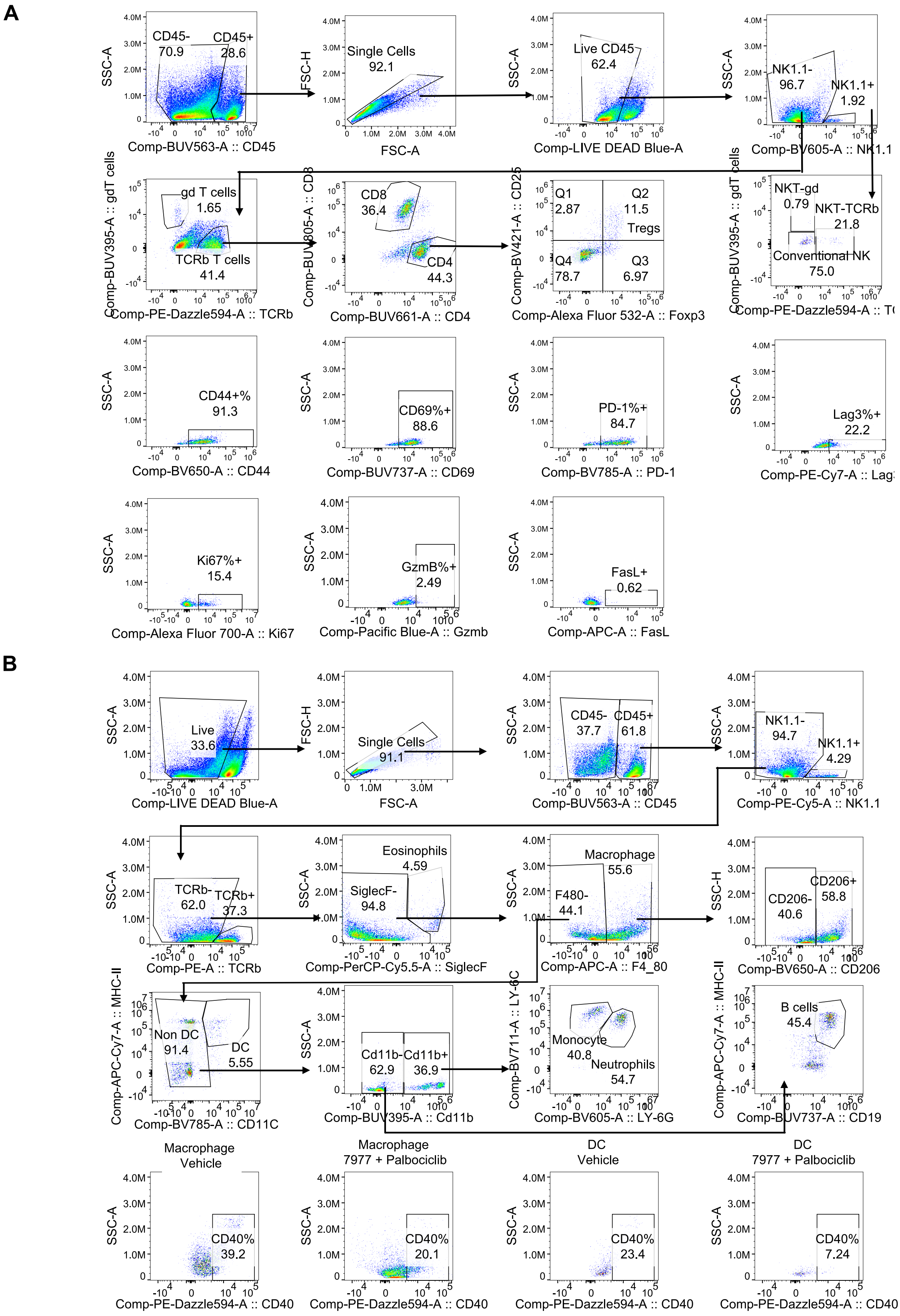
A
CD45- 70.9
CD45+ 28.6
Comp-BUV563-A :: CD45
Single Cells 92.1
FSC-A
FSC-H
Live CD45 62.4
Comp-LIVE DEAD Blue-A
NK1.1- 96.7
NK1.1+ 1.92
Comp-BV605-A :: NK1.1
gd T cells 1.65
TCRb T cells 41.4
Comp-PE-Dazzle594-A :: TCRb
Comp-BUV395-A :: gdT cells
CD8 36.4
CD4 44.3
Comp-BUV661-A :: CD4
Q1 2.87
Q2 11.5
Tregs
Q4 78.7
Q3 6.97
Comp-Alexa Fluor 532-A :: Foxp3
Comp-BV421-A :: CD25
NKT-gd 0.79
NKT-TCRb 21.8
Conventional NK 75.0
CD44+% 91.3
Comp-BV650-A :: CD44
CD69%+ 88.6
Comp-BUV737-A :: CD69
PD-1%+ 84.7
Comp-BV785-A :: PD-1
Lag3%+ 22.2
Ki67%+ 15.4
Comp-Alexa Fluor 700-A :: Ki67
GzmB%+ 2.49
Comp-Pacific Blue-A :: Gzmb
FasL+ 0.62
Comp-APC-A :: FasL
SSC-A
B
Live 33.6
Comp-LIVE DEAD Blue-A
Single Cells 91.1
CD45- 37.7
CD45+ 61.8
Comp-BUV563-A :: CD45
NK1.1- 94.7
NK1.1+ 4.29
Comp-PE-Cy5-A :: NK1.1
TCRb- 62.0
TCRb+ 37.3
Comp-PE-A :: TCRb
Eosinophils 4.59
SiglecF- 94.8
Comp-PerCP-Cy5.5-A :: SiglecF
Macrophage 55.6
F480- 44.1
Comp-APC-A :: F4_80
CD206- 40.6
CD206+ 58.8
Comp-BV650-A :: CD206
SSC-H
Non DC 91.4
DC 5.55
Comp-BV785-A :: CD11C
Comp-APC-Cy7-A :: MHC-II
Cd11b- 62.9
Cd11b+ 36.9
Comp-BUV395-A :: Cd11b
Monocyte 40.8
Neutrophils 54.7
Comp-BV605-A :: LY-6G
Comp-BV711-A :: LY-6C
B cells 45.4
Comp-BUV737-A :: CD19
Macrophage Vehicle
CD40% 39.2
Macrophage 7977 + Palbociclib
CD40% 20.1
DC Vehicle
CD40% 23.4
DC 7977 + Palbociclib
CD40% 7.24
Comp-PE-Dazzle594-A :: CD40

**Supplementary Figure S8. Gating strategy for flow cytometry analysis in Fig 3A-B and Sup Fig 4A-N, Q-S.**
**(A)** Gating strategy for T and NK cell activation and exhaustion panel
**(B)** Gating strategy for all immune cell subset identification and CD40 expression panel.
